# Nanosystem Delivers Senescence Activators and Immunomodulators to Combat Liver Cancer

**DOI:** 10.1002/advs.202308310

**Published:** 2024-03-23

**Authors:** Ke Gong, Juyang Jiao, Zhihua Wu, Quan Wang, Jinghan Liao, Yi Duan, Jiangtao Lin, Jian Yu, Ying Sun, Yong Zhang, Yourong Duan

**Affiliations:** ^1^ State Key Laboratory of Systems Medicine for Cancer Shanghai Cancer Institute Renji Hospital School of Medicine Shanghai Jiao Tong University Shanghai 200032 P. R. China; ^2^ Department of Bone and Joint Surgery Department of Orthopedics Renji Hospital School of Medicine Shanghai Jiao Tong University Shanghai 200001 P. R. China; ^3^ School of Chemistry and Chemical Engineering Shanghai Key Laboratory of Electrical Insulation and Thermal Aging Shanghai Jiao Tong University Shanghai 200240 P. R. China

**Keywords:** combination therapy, dual‐target, immunotherapy, senescence, sequential release

## Abstract

CD47 blockade has emerged as a promising immunotherapy against liver cancer. However, the optimization of its antitumor effectiveness using efficient drug delivery systems or combinations of therapeutic agents remains largely incomplete. Here, patients with liver cancer co‐expressing CD47 and CDC7 (cell division cycle 7, a negative senescence‐related gene) are found to have the worst prognosis. Moreover, CD47 is highly expressed, and senescence is inhibited after the development of chemoresistance, suggesting that combination therapy targeting CD47 and CDC7 to inhibit CD47 and induce senescence may be a promising strategy for liver cancer. The efficacy of intravenously administered CDC7 and CD47 inhibitors is limited by low uptake and short circulation times. Here, inhibitors are coloaded into a dual‐targeted nanosystem. The sequential release of the inhibitors from the nanosystem under acidic conditions first induces cellular senescence and then promotes immune responses. In an in situ liver cancer mouse model and a chemotherapy‐resistant mouse model, the nanosystem effectively inhibited tumor growth by 90.33% and 85.15%, respectively. Overall, the nanosystem in this work achieved the sequential release of CDC7 and CD47 inhibitors in situ to trigger senescence and induce immunotherapy, effectively combating liver cancer and overcoming chemoresistance.

## Graphical abstract

The sequential release of a senescence inducer (CDC7 inhibitor) and a CD47 inhibitor from the dual‐targeting nanosystem in situ induced senescence and antitumor immune responses to aganist liver cancer and overcome chemoresistance.

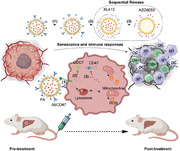

## 1 Introduction

Hepatocellular carcinoma (HCC) is one of the most common types of primary cancer and is the fourth leading cause of cancer‐related mortality worldwide.^[^ ^1^ ^]^ Immunotherapies based on tumor antigen‐targeting antibodies and immunomodulatory antibodies are emerging approaches that may improve HCC treatment outcomes. CD47 is an innate inhibitory immune checkpoint that activates the “Don't eat me” signal by binding to the N‐terminus of SIRP‐α (signal regulatory protein α), a signal regulatory protein on immune cells.^[^ ^2^, ^3^, ^4^ ^]^ CD47 is highly expressed on HCC cells, allowing them to evade the immune system.^[^ ^5^ ^]^ Studies have shown that blocking the CD47/SIRP‐α signaling axis can promote the phagocytosis and polarization of M1 macrophages.^[^ ^6^ ^]^ Researchers have found that inhibiting CD47 can be effective in treating cancer.^[^ ^7^, ^8^, ^9^ ^]^ The effectiveness of immunotherapy depends on the immune status of patients with HCC, and immunotherapy often needs to be combined with other treatments.^[^ ^10^, ^11^ ^]^ Combination treatment with CD47 monoclonal antibodies and PD‐1 monoclonal antibodies effectively inhibits the cancer.^[^ ^12^ ^]^ Combined targeting of CD47 and glypican‐3 is efficacious against dual antigen‐expressing HCC.^[^ ^13^ ^]^ The strategies mentioned above illustrate that anti‐CD47‐based combination therapies offer promise for the clinical treatment of HCC. However, only a limited subgroup of patients benefit from these treatments. Thus, more efficacious anti‐CD47‐based combination treatments are needed for the treatment of a larger proportion of HCC patients.

Research has shown that inducing senescence in cancer cells promotes antitumor immune responses.^[^ ^14^ ^]^ Cellular senescence is usually a response to intrinsic or extrinsic cellular stresses such as DNA damage.^[^ ^15^ ^]^ Senescence is often considered a protective tumor suppressor because it prevents cancer cell proliferation and progression.^[^ ^16^ ^]^ Growth‐stunted cancer cells, however, can escape senescence and produce more aggressive tambours.^[^ ^14^ ^]^ The studies mentioned above indicate that we can treat HCC by inducing senescence. Inhibiting CDC7 prevents the start of DNA replication and promotes the apoptosis of cancer cells.^[^ ^17^, ^18^ ^]^ Moreover, CDC7 inhibition promotes HCC cell senescence,^[^ ^19^ ^]^ indicating that CDC7 inhibitors can act as senescence activators. Therefore, combination therapy involving both CD47 inhibitors and senescence activators (CDC7 inhibitors) is a promising strategy for HCC treatment.

Traditional drugs are rapidly metabolized, cause systemic toxicity, and are insufficiently effective in the treatment of tumors.^[^ ^20^ ^]^ Moreover, for multidrug combinations, the frequency of dosing and the intervals between doses need to be addressed, and numerous doses are often needed, which imposes a certain burden on both patients and doctors.^[^ ^21^, ^22^ ^]^ Nanoparticles (NPs) are able to codeliver lipid‐soluble and water‐soluble drugs.^[^ ^23^, ^24^ ^]^ NPs are stable in serum, thus prolonging the drug circulation time, increasing the accumulation of drugs at tumor sites through enhanced permeability and retention (EPR) effects, reducing toxic side effects, and decreasing the frequency of drug administration.^[^ ^25^, ^26^, ^27^ ^]^

Because the EPR effect is limited, to improve the active targeting of NPs to tumors, we prepared NPs modified with a CD47 antibody (AbCD47). Because CD47 is highly expressed on HCC,^[^ ^5^ ^]^ AbCD47 can target and block CD47 on the surface of tumor cells to disable the “Don't eat me” signal. However, tumor heterogeneity causes the amount of CD47 to vary from cell to cell,^[^ ^28^ ^]^ leading to targeting failure. To ensure adequate tumors targeting, a second target needs to be added. Folate receptor 1 (FOLR1), was originally identified as a folic acid (FA)‐binding protein,^[^ ^29^, ^30^ ^]^ has been shown to be barely expressed in normal cells but widely overexpressed in solid tumor cells, including HCC cells.^[^ ^31^ ^]^ These studies suggest that FOLR1 may serve as a specific tumor antigen for HCC cells. Therefore, we utilized FA as the second targeting moiety to target HCC cells by binding with FOLR1 to remedy the absence of target genes due to tumor heterogeneity.

**Scheme 1:**
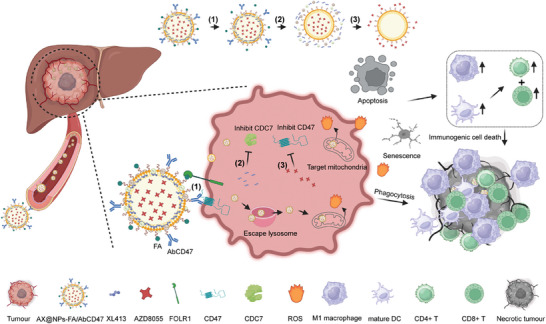
Schematic diagram of the use of AX@NPs‐FA/AbCD47 for the treatment of liver cancer to induce senescence and antitumor immune responses. The dual‐targeting AX@NPs‐FA/AbCD47 gradually accumulated at the tumor site and were then taken up by HCC cells. Next, the NPs escaped the lysosome and then sequentially released the drugs XL413 and AZD8055 within the acidic tumor niche to block CDC7 and CD47, inducing apoptosis, cellular senescence, and antitumor immune responses for the treatment of liver cancer. In addition, the NPs target mitochondria and induce the production of ROS, thereby destroying the tumor microenvironment and enhancing the antitumor immune response.

We constructed dual‐targeting calcium‐phosphorus NPs coloaded with CD47 and CDC7 inhibitors using 1,2‐distearoyl‐sn‐glycero‐3‐phosphoethanolamine‐N‐[amino(polyethylene glycol)−2000] (DSPE‐PEG2000‐NH_2_), which is a main component of cell membranes, and PEG2000, which has been approved by the FDA for use in humans.^[^ ^32^ ^]^ Calcium‐phosphorus NPs with pH‐responsive properties ensure the long‐term sequential release of drugs into tumors.^[^ ^33^ ^]^ Compared to conventional drug delivery, our nanodelivery system is expected to provide a new therapeutic option for liver cancer treatment due to its dual‐targeting and combined induction of senescence and immunomodulation. (**Scheme** **1**)

## 2 Results

### 2.1 CD47 and CDC7 Overexpression Predicted Poor Prognosis in Patients with HCC

**Figure 1:**
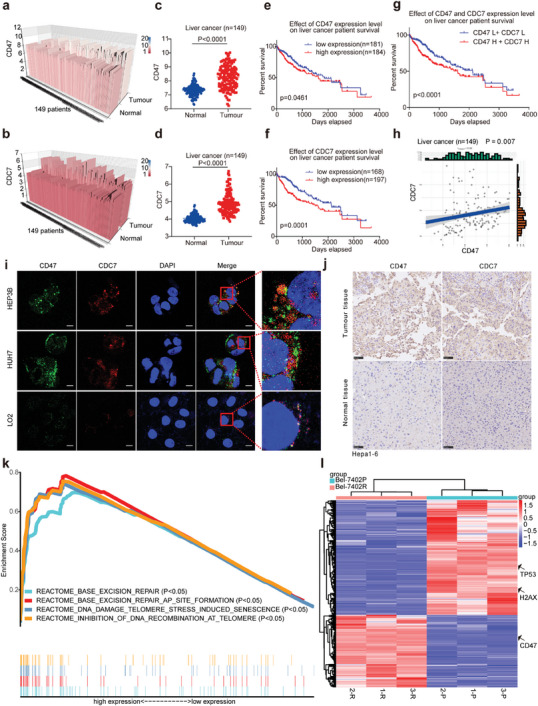
CD47 and CDC7 overexpression predicted poor prognosis in patients with HCC. a,b) The expression of CD47 and CDC7 in 149 liver cancer patients (tumor tissue and adjacent normal tissue) was analyzed in GSE76297. c,d) Statistical analysis of CD47 and CDC7 expression in HCC. e,f) Analysis of the TCGA database to reveal the effect of CD47 and CDC7 expression on survival time in patients with HCC. g) Effects of CD47 and CDC7 expression on the prognosis of patients with HCC. L indicates low expression, and H indicates high expression. h) Correlation analysis of CD47 and CDC7 in 149 liver cancer patients. i) Immunofluorescence analysis of CD47 and CDC7 expression in HCC cells (HEP3B and HUH7) and normal hepatocytes (LO2). Green represents CD47, red represents CDC7, and blue represents the nucleus. j) Immunohistochemical results of CD47 and CDC7 in Hepa1‐6 tumor‐bearing mice. k) Pathway enrichment analysis of Bel‐7402P and Bel‐7402R using GSEA software. l) Sequencing results for the differentially expressed genes in Bel‐7402P (1‐P, 2‐P, and 3‐P) and Bel‐7402R (1‐R, 2‐R, and 3‐R). The data are present as the mean ± SEM, and a p‐value less than 0.05 was considered to indicate statistical significance.

By screening 149 pairs of liver cancer specimens in the GEO database GSE76297 (**Figure** **1**a,b), we found that CD47 and CDC7 were highly expressed in cancer tissues (Figure 1c,d). An additional analysis of five pairs of liver cancer specimens (GSE146049) confirmed that CD47 and CDC7 were highly expressed in HCC tissues (Figure S1, Supporting Information). These results suggest that CD47 and CDC7 have clinical diagnostic value. To further evaluate the clinical diagnostic value of CD47 and CDC7, we analyzed the TCGA database and divided 365 patients with HCC into high‐expression and low‐expression groups according to the fragments per kilobase million (FPKM) values of CD47 and CDC7. Figure 1e,f shows that high expression levels of CD47 or CDC7 predicted poor prognosis in HCC patients. In addition, with the HCC patients with the worst prognosis (p≤0.0001) had high expression of both CD47 and CDC7 (Figure 1g). These results suggest that CD47 and CDC7 are promising therapeutic targets for HCC. Figure 1h and Figure S2 (Supporting Information) show that the CD47 and CDC7 expression levels in HCC patients were positively correlated, suggesting that these proteins may have a synergistic tumor‐promoting effect. The expression of major histocompatibility complex I (MHC I) determines the immune antitumor response.^[^ ^34^ ^]^ Hence, we analyzed the sequencing results of 149 patients, and Figure S3 (Supporting Information) shows that the expression of MHC class I chain‐related proteins A and B (MICA and MICB) was significantly upregulated in HCC patients. This result suggested that HCC tumor cells are sensitive to immunotherapy. To further verify the upregulated expression of CD47 and CDC7 in HCC, we compared the HCC cell lines HEP3B and HUH7 with the normal hepatocyte cell line LO2. Figure 1i shows that CD47 and CDC7 were significantly upregulated in HCC cells compared to normal hepatocytes. We also examined CD47 and CDC7 expression in Hepa1‐6 in situ HCC model mice, and Figure 1j shows that CD47 and CDC7 were both more highly expressed in tumor tissue than in adjacent normal liver tissue. These results suggest that CD47 and CDC7 may be potential targets for the clinical treatment of HCC. Moreover, we generated a chemoresistant HCC cell line, Bel‐7402R, from the parental cell line Bel‐7402P. We performed RNA sequencing of Bel‐7402R and Bel‐7402P cells and demonstrated that cell senescence and DNA damage repair‐related pathways were significantly enriched after the development of drug resistance (Figure 1k; Figure S4, Supporting Information), and CD47 was upregulated in the drug‐resistant cell line compared to the sensitive cell line (Figure 1l). Moreover, the expression of senescence‐associated genes (TP53 and H2AX) was downregulated in drug‐resistant cells (Figure 1l). These results revealed the downregulation of cellular senescence‐associated genes and upregulation of CD47 after the development of drug resistance in HCC, and CDC7 is negatively correlated with senescence, suggesting that the inhibition of CDC7 and CD47 may be able to overcome chemoresistance in HCC.

### 2.2 Downregulation of CD47 and CDC7 Synergistically Induced Cellular Senescence and Phagocytosis In Vitro

**Figure 2:**
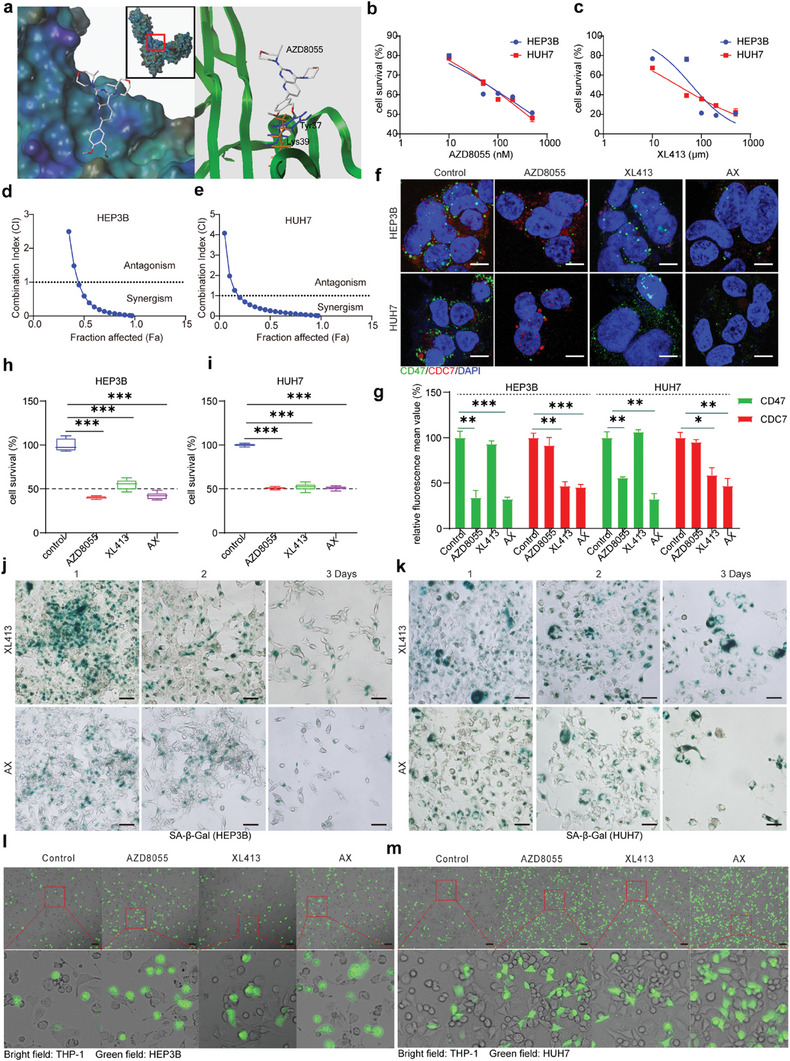
Downregulation of CD47 and CDC7 synergistically induced senescence and phagocytosis in HCC cells. a) Left: Overview of the molecular modelling of AZD8055 binding to CD47 (PDB code: 2VSC); Right: The inside binding residues, Lys39 (orange) and Tyr37 (blue). Yellow dashed lines, hydrogen bonds between AZD8055 and CD47. b,c) The CCK8 assay was used to analyse the IC50 values of AZD8055 and XL413 against HEP3B and HUH7 cells. d,e) CompuSyn software was used to determine the synergistic effect of AZD8055 and XL413 on HEP3B and HUH7 cells, and the Chou‐Talalay method was used (combination index: CI < 1 indicated synergistic effect). f,g) Immunofluorescence analysis of CD47 and CDC7 expression in cells (HEP3B and HUH7) treated with drugs for 48 h (HEP3B: control, 0.5 µm AZD8055, 60 µm XL413, 0.08 µm AZD8055 ± 9.5 µm XL413). HUH7: control, 0.4 µm AZD8055, 30 µm XL413, 0.03 µm AZD8055 ± 2.1 µm XL413). Green represents CD47, red represents CDC7, and blue represents the nucleus, n = 3, mean ± SEM, ^*^ *p* ≤0.05, ^**^ *p* ≤0.01, ^***^ *p* ≤0.001. h,i) A CCK8 assay was used to determine the viability of HEP3B and HUH7 after incubation with certain drugs for 72 h (HEP3B: control, 0.5 µM AZD8055, 60 µm XL413, 0.08 µm AZD8055 + 9.5 µm XL413; HUH7: control, 0.4 µm AZD8055, 30 µm XL413, 0.03 µm AZD8055 + 2.1 µm XL413), n = 5, mean ± SEM, ^***^ *p* ≤0.001. j,k) β‐Galactosidase staining was used to determine cellular senescence in HEP3B and HUH7 cells after treatment with drugs for 3 days (HEP3B: control, 0.5 µm AZD8055, 60 µm XL413, 0.08 µm AZD8055 + 9.5 µm XL413;. HUH7: control, 0.4 µm AZD8055, 30 µm XL413, 0.03 µm AZD8055 + 2.1 µm XL413). l,m) THP‐1 cells were induced to become macrophages and cocultured with HCC cells with drugs for 1 h. (HEP3B: control, 0.5 µm AZD8055, 60 µm XL413, 0.08 µm AZD8055 + 9.5 µm XL413; HUH7: control, 0.4 µm AZD8055, 30 µm XL413, 0.03 µm AZD8055 + 2.1 µm XL413) The phagocytosis of HCC cells by macrophages was observed. Green represents HCC cells; the bright field represents THP‐1 cells.

Studies have shown that CD47 signaling is mediated by the activation of mTOR (mammalian target of rapamycin).^[^ ^35^, ^36^ ^]^ Hence, we predicted that inhibiting mTOR could suppress CD47. AZD8055 is a mTOR inhibitor that has been investigated in clinical trials.^[^ ^37^, ^38^ ^]^ We used computer‐simulated docking to evaluate the binding of the CD47 protein to AZD8055. The findings indicated that AZD8055 effectively docks at Lys39 (lysine 39, depicted in orange) and Tyr37 (tyrosine 37, depicted in blue) in CD47, by forming hydrogen bonds, as demonstrated in **Figure** **2**a. Furthermore, treating the HEP3B and HUH7 cell lines with AD8055, elicited a notable reduction in CD47 expression, as illustrated in Figure S5 (Supporting Information). This finding suggested that AZD8055 has the potential to serve as a potent small‐molecule inhibitor of CD47. XL413 is a selective inhibitor of CDC7. Figure 2b,c indicates that AZD8055 and XL413 effectively killed HCC cells. The concentration that inhibited cell proliferation by 50% relative to the control (IC50) was calculated using GraphPad Prism 8.0 (HEP3B: AZD8055 0.5 µm, XL413 60 µm; HUH7: AZD8055 0.4 µm, XL413 30 µm). Synergy was detected following the Chou‐Talalay method (combination index: CI < 1 indicates synergy)^[^ ^39^ ^]^ using CompuSyn software (Ver.1.0). The synergistic index of HEP3B and HUH7 was less than 1 when the fraction affected (Fa) was 0.5 (Figure 2d,e). Thus, we determined the synergistic ratio of the two drugs in HCC cells at the Fa value of 0.5 (HEP3B: 0.08 µm AZD8055 ± 9.5 µm XL413; HUH7: 0.03 µm AZD8055 ± 2.1 µm XL413). Figure 2f,g shows that AZD8055 and XL413 downregulated CD47 and CDC7, respectively. We verified the synergistic effect of the two drugs with CCK8 assays, and the results showed that the drug combination had significant cytotoxicity, and the combination drug dose was much lower than the single drug doses (Figure 2h,i). Studies have indicated that XL413 can induce cellular senescence.^[^ ^19^ ^]^ As shown in Figure 2j,k, XL413 promoted cellular senescence, and when combined with AZD8055, it also promoted cellular senescence. A certain degree of cellular senescence induces apoptosis,^[^ ^40^ ^]^ and AZD8055 can promote apoptosis. Figure S6 (Supporting Information) shows that AZD8055 and XL413 promoted apoptosis in HCC cells either individually or in combination. The downregulation of CD47 on cells disengages the “Don't eat me” signal and promotes phagocytosis.^[^ ^41^ ^]^ Figure 2l,m and Figure S7 (Supporting Information) show that AZD8055 significantly promoted phagocytosis; in addition, XL413 promoted phagocytosis, which we speculate is because the drugs induced cellular senescence or apoptosis and macrophages perform phagocytosis of senescent and damaged cells (including apoptotic cells). In summary, we found that AZD8055 downregulated CD47 expression and synergized with the CDC7 inhibitor XL413 to promote cellular senescence and phagocytosis.

### 2.3 Formation and Characterization of NPs

AbCD47 can target and block CD47 on the surface of tumor cells to disable the “Don't eat me” signal. However, tumor heterogeneity causes the amount of CD47 to vary from cell to cell,^[^ ^28^ ^]^ leading to targeting failure. To ensure tumor targeting, another target needs to be added. We found that FOLR1 was highly expressed in HCC tissues (Figure S8a,b, Supporting Information), and TCGA database analysis indicated that high FOLR1 expression predicted poor patient prognosis (Figure S8c, Supporting Information). FOLR1 was originally identified as a folic acid (FA)‐binding protein^[^ ^29^, ^30^ ^]^ and is barely expressed in normal cells.^[^ ^31^ ^]^ Hence, FA was utilized as an auxiliary targeting molecule to improve targeting.

**Figure 3:**
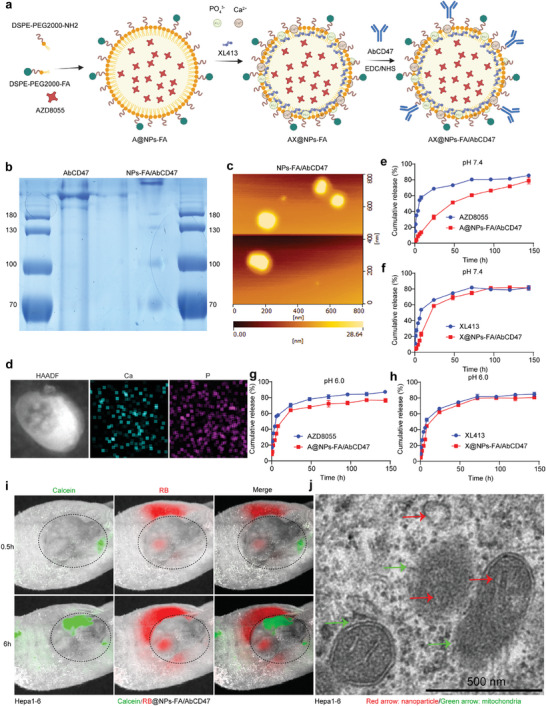
Formation and characterization of NPs. a) Synthetic procedure diagram of AX@NPs‐FA/AbCD47. b) Detection of AbCD47 modified NPs by SDS‒PAGE gel; the amount of free AbCD47 was 4 µg, and the NPs‐FA/AbCD47 contained the same dose of AbCD47. c) Morphology of the NPs‐FA/AbCD47 shown by AFM. d) Calcium and phosphorus in the NPs were elementally analyzed by field emission TEM. HAADF: high‐angle annular dark field. e,f) Release of AZD8055 and XL413 at pH 7.4. g,h) Release of AZD8055 and XL413 at pH 6.0. i) Live imaging of small animals to visualize the release of nanodrugs (Calcein/RB@NPs‐FA/AbCD47) in animals. Red represents rhodamine inside the NPs; green represents calcein inside the shell. j) TEM images of NPs targeting mitochondria. Red arrows indicate NPs, and green arrows indicate mitochondria.

**Figure** **3**a illustrates the formation process of AX@NPs‐FA/AbCD47. Figure S9a (Supporting Information) shows that the NPs were modified with AbCD47. The free AbCD47 moved through the gel faster than the AbCD47 attached to the NPs, and SDS‒PAGE indicated that the NPs were successfully modified with AbCD47 (Figure 3b). NPs‐FA/AbCD47 were photographed by atomic force microscopy (AFM), which showed a ring of small particles circling the NPs, forming a “shell‐core” structure (Figure 3c). Figure 3d shows the adsorption of calcium and phosphorus on the NP surface. Figure S9b–e (Supporting Information) shows that AX@NPs‐FA/AbCD47 had a diameter of 122.5 ± 2.37 nm and a PDI of 0.235 ± 0.013. After incubation in 5% FBS solution (pH 7.4) at 37 °C for 7 days, the sizes and PDI of the NPs changed very little, indicating that the NPs remained stable in storage. At pH 7.4, these NPs were negatively charged (−10.3 mV), which prevents the adsorption of albumin and favors circulation in the blood,^[^ ^42^ ^]^ whereas at pH 6.0, they were positively charged (0.00705 mV), which facilitates uptake by tumor cells.^[^ ^43^ ^]^ The encapsulation efficiency (EE%) values for AZD8055 and XL413 were 83.5% and 75.1%, respectively. We mimicked in vivo drug release conditions in vitro, and the results indicated that AZD8055 and XL413 were released faster from NPs at pH 6.0 than at pH 7.4. Moreover, XL413 was released from the NPs earlier than AZD8055 (Figure 3e–h). Calcein/RB@NPs‐FA/AbCD47 with an outer layer of adsorbed calcein and an inner layer of rhodamine (RB) were injected into mice bearing Hepa1‐6 subcutaneous tumors via the tail vein, and small animal live imaging revealed changes in fluorescence with time. Little drug enrichment was observed at the tumor site before 0.5 h. After 6 h, the drugs accumulated at the tumor site, and the drug in the outer layer (calcein: green fluorescence) was released first, while the drug in the inner layer (rhodamine: red fluorescence) was released later (Figure 3i). These results confirmed that calcium and phosphorus were sensitive to pH changes, guaranteeing the sequential release of AZD8055 and XL413 in the acidic tumor niche. Moreover, the TEM results suggested that the NPs targeted mitochondria in the tumor cells (Figure 3j). Figure S10 (Supporting Information) implies that the NPs do not cause haemolysis, indicating suitable biosafety. To explore the CD47 targeting ability of the CD47 antibody, we incubated free RB and RB NPs with HCC cells. Figure S11 (Supporting Information) shows that RB@NPs‐FA/AbCD47 were taken up by HCC cells much more frequently than free RB, which indicated that the FA and CD47 antibodies modifications promoted NP uptake by HCC cells. Figure S12 (Supporting Information) shows that RB@NPs‐FA/AbCD47 selectively targeted HCC cells but barely targeted erythrocytes. Escaping the degradation and clearance of the lysosome is a prerequisite for the effective delivery of a drug by NPs and the ability to exert its effects.^[^ ^44^ ^]^ Partial phagocytosis of NPs by lysosomes was observed at 1 h, and after incubation for 4 h, most of the NPs had escaped lysosomal degradation (Figure S13, Supporting Information), suggesting that the NPs could effectively deliver drugs into cells. In summary, the NPs released drugs in a sequential manner. Moreover, the NPs were readily taken up by HCC cells and evaded lysosomal clearance.

### 2.4 Mitochondrion‐Targeted AX@NPs‐FA/AbCD47 Increased Cellular Senescence and Phagocytosis In Vitro

**Figure 4:**
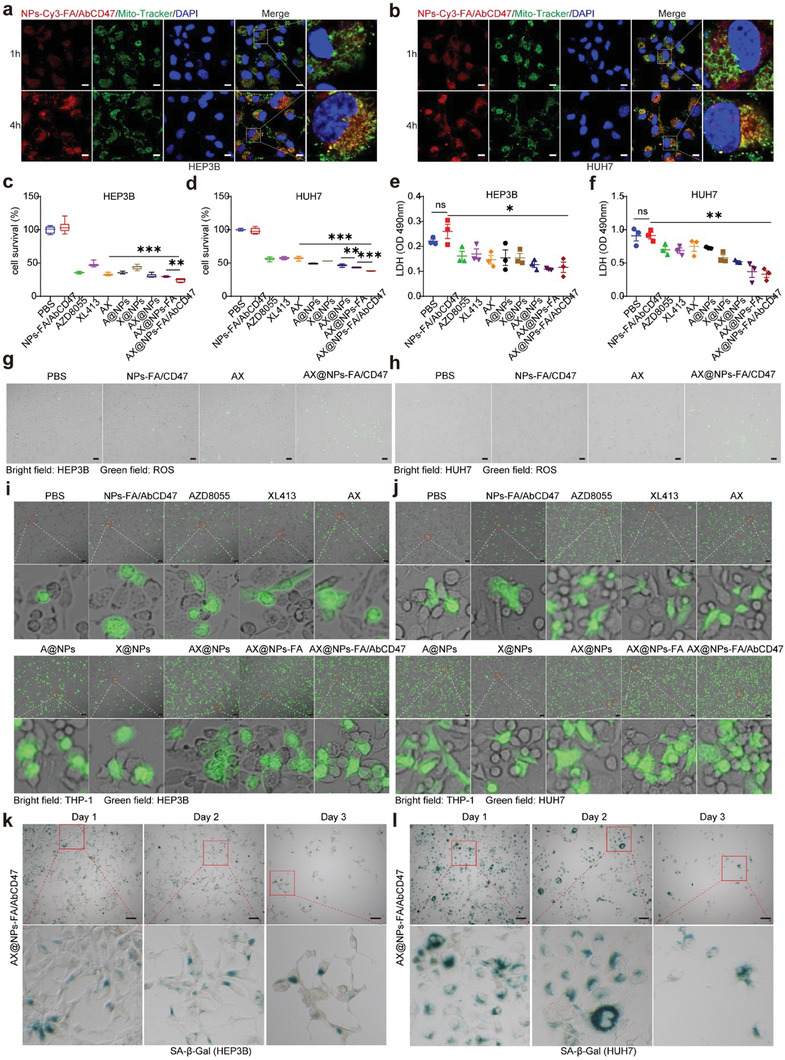
Mitochondrion‐targeted AX@NPs‐FA/AbCD47 increased cellular senescence and phagocytosis in vitro. a,b) Fluorescence micrographs of NPs targeting the mitochondria of HCC cells at 1 h and 4 h. Red represents NPs‐Cy3‐FA/AbCD47, green represents the mitochondria, and blue represents the nucleus. Red and green indicate that the NPs did not target the mitochondria, whereas red and green combined with orange indicate that the NPs did target the mitochondria. c,d) A cytotoxicity assay was used to verify the killing effect of the nanodrugs on HEP3B and HUH7 cells, n = 6, mean ± SEM, ^**^ *p* ≤ 0.01, ^***^ *p* ≤0.001. e,f) Treatment of HCC cells with the nanodrugs and measurement of LDH levels released from cells, n = 3, mean ± SEM, ns means indicates no significant difference, ^*^ *p* ≤0.05, ^**^ *p* ≤0.01. g,h) Detection of intracellular ROS production by fluorescence microscopy after the nanodrug treatment of HCC cells. Green represents ROS; the bright field represents HCC cells. i,j) THP‐1 cells were induced to become macrophages and cocultured with HCC cells for 1 h. The phagocytosis of HCC cells by macrophages was observed. Green represents HCC cells; bright field represents THP‐1 cells. k,l) β‐Galactosidase staining to observe AX@NPs‐FA/AbCD47‐induced senescence in HCC cells.

One study reported that DSPE‐PEG2000‐NH_2_ can participate in mitochondrial targeting.^[^ ^45^ ^]^ We treated HCC cells with NPs‐Cy3‐FA/AbCD47. **Figure** **4**a,b shows that at 1 h, the NPs did not target the mitochondria; presumably, they were taken up by lysosomes and had not yet escaped. At 4 h, the NPs had targeted the mitochondria. Figure 4c,d shows that the nanodrugs were more effective at killing HCC cells. AX@NPs‐FA/AbCD47 significantly decreased the LDH level (Figure 4e,f). Mitochondria are the primary source of cellular reactive oxygen species (ROS),^[^ ^46^ ^]^ and we examined ROS levels in HCC cells. Figure 4g,h and Figure S14 (Supporting Information) show that treatment with AX@NPs‐FA/AbCD47 elicited a large amount of intracellular ROS production. The increased ROS production indicates damage to the HCC cells. Our previous study showed that AZD8055 could act as a small molecule inhibitor of CD47, and that downregulation of CD47 could promote the phagocytosis of tumor cells by macrophages. Figure 4i,j and Figure S15 (Supporting Information) show that the nanodrug significantly promoted the phagocytosis of HCC cells by macrophages. As XL413 promoted tumor cell senescence, we treated HCC cells with AX@NPs‐FA/AbCD47, and Figure 4k,l shows that AX@NPs‐FA/AbCD47 induced HCC cell senescence at an early stage. These results confirm that AX@NPs‐FA/AbCD47 can trigger HCC cell senescence and promote phagocytosis, thereby killing tumor cells.

### 2.5 AX@NPs‐FA/AbCD47 Alleviated Liver Cancer and Overcame Chemoresistance In Vivo

**Figure 5:**
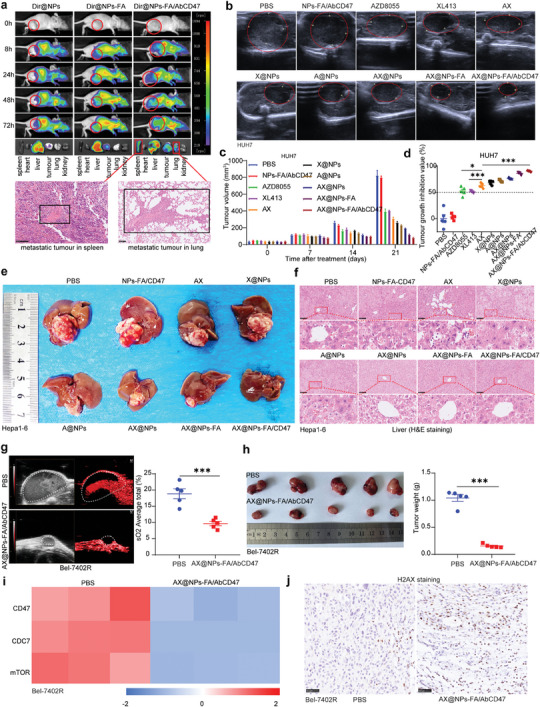
AX@NPs‐FA/AbCD47 alleviated liver cancer and overcame chemoresistance in vivo. a) An in vivo imaging system was used to detect the targeting of NPs (HUH7 subcutaneous tumor in nude mice). b) In situ ultrasound imaging results of HUH7 liver cancer in different treatment groups in nude mice for different periods. c) The tumor growth curve of HUH7 liver cancer in nude mice, n = 5. d) The tumor growth inhibition value after treatment, n = 5, mean ± SEM, ^*^ *p* ≤0.05, ^***^ *p* ≤0.001. e) Ex vivo anatomical images of different treatment groups of C57BL/6 mice (Hepa1‐6). f) H&E staining of liver tissue within 0.5 cm of the cancer after treatment of Hepa1‐6 in situ HCC. g) Intratumoral blood oxygenation observed by photoacoustic imaging of subcutaneous Bel‐7402 tumors in mice after treatment and the statistical results of intratumoral blood oxygenation, n = 5, mean ± SEM, ^***^ *p* ≤0.001. h) Photographs of tumors in mice after treatment and statistical analysis of tumor weight in mice, n = 5, mean ± SEM, ^***^ *p* ≤0.001; i) Heatmap of CD47, CDC7, and mTOR expression in tumors after treatment with PBS and AX@NPs‐FA/AbCD47. j) H2AX staining in Bel‐7402R tumor‐bearing mice after treatment.

A prerequisite for NP treatment of tumors in vivo is effective delivery to the tumor.^[^ ^47^ ^]^ We subcutaneously inoculated HUH7 cells into nude mice and used an in vivo imaging system to evaluate the tumor‐targeting ability of the NPs. **Figures** **5**a and S16 (Supporting Information) show that Dir@NPs‐FA/AbCD47 had the best targeting ability, and a large amount of fluorescence was still present at the tumor site at 72 h. In addition, when the subcutaneous tumor in the Dir@NPs‐FA/AbCD47 group metastasized to the spleen and lung, the NPs effectively targeted this metastasis site, while the other groups with metastasized tumors showed no fluorescence in the spleen and lung. We subcutaneously inoculated HUH7 liver cancer cells into the liver, as shown in Figure S17a (Supporting Information). Figure S17b (Supporting Information) shows the therapeutic period of the mice. Tumor growth was recorded in vivo with small animal ultrasound equipment, and Figure 5b,c revealed that the AX@NPs‐FA/AbCD47 group had the best treatment effect, and the HUH7 tumor growth inhibition value reached 90.33% (Figure 5d). In addition, the growth status of the mice in the AX@NPs‐FA/AbCD47‐treated group was better than that of the mice in the control group (Figure S18, Supporting Information). To further verify the effectiveness of AX@NPs‐FA/AbCD47, we established a Hepa1‐6 HCC in situ C57BL/6 mouse model, as shown in Figure S17a (Supporting Information). The treatment period is shown in Figure S17b (Supporting Information), and at the end of the treatment, the C57BL/6 mice were sacrificed and dissected. The results in Figure 5e suggest that treatment with AX@NPs‐FA/AbCD47 had the best treatment effect. In addition, AX@NPs‐FA/AbCD47 effectively inhibited the infiltration of HCC cells into normal liver tissues adjacent (<0.5 cm) to the cancerous tissues (Figure 5f). To observe the therapeutic effect of AX@NPs‐FA/AbCD47 on drug‐resistant HCC cells, we established drug‐resistant Bel‐7402R subcutaneous tumors. AX@NPs‐FA/AbCD47 markedly inhibited the intratumoral blood supply (Figure 5g), and Figure 5h shows that the NPs clearly inhibited the growth of the drug‐resistant HCC tumors (tumor growth inhibition value: 85.15%). In addition, the CD47, CDC7 and mTOR levels in Bel‐7402R tumors were significantly reduced after treatment (Figure 5i). Furthermore, treatment with AX@NPs‐FA/AbCD47 promoted the accumulation of the senescence‐related protein H2AX in Bel‐7402R tumors, suggesting that the nanomedicine effectively induced senescence in drug‐resistant tumors (Figure 5j; Figure S19, Supporting Information). The above results demonstrated that AX@NPs‐FA/AbCD47 were effective in treating HCC and drug‐resistant HCC.

### 2.6 AX@NPs‐FA/AbCD47 Downregulated CD47 and CDC7 to Inhibit Tumor Growth and Promote Cellular Senescence In Vivo

**Figure 6:**
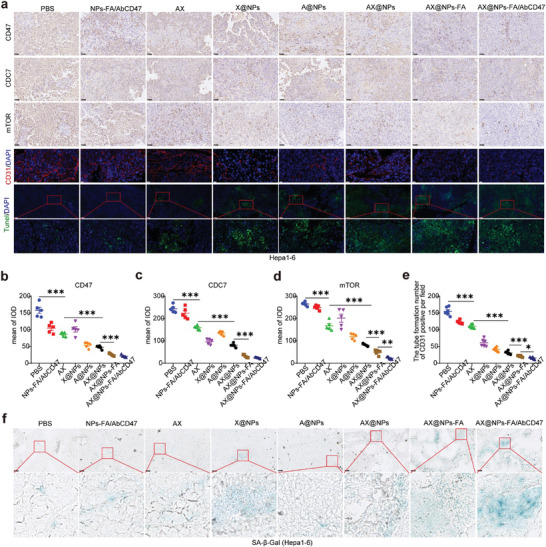
AX@NPs‐FA/AbCD47 promoted cellular senescence and inhibited tumor growth by downregulating CD47 and CDC7. a) The expression of CD47, CDC7, mTOR, and CD31 in Hepa1‐6 tumors from each treatment group and TUNEL staining of Hepa1‐6 tumors from C57BL/6 mice in different treatment groups. Green represents TUNEL staining, and blue represents nuclei. b) Statistical analysis of CD47 expression in various treatment groups. c) Statistical analysis of CDC7 expression in various treatment groups. d) Statistical analysis of mTOR expression in various treatment groups; e) Statistical analysis of CD31 expression in various treatment groups. f) Staining for SA‐β‐Gal in Hepa1‐6 tumors from each treatment group. n = 5, mean ± SEM, ^*^ *p* <0.05, ^**^ *p* <0.01, ^***^ *p* <0.001.

We analyzed the expression levels of CD47, CDC7, and mTOR in the tumors of C57BL/6 mice after different treatments, and **Figure** **6**a shows that AX@NPs‐FA/AbCD47 significantly downregulated CD47, CDC7, and mTOR to inhibit angiogenesis and promote apoptosis (Figure 6b–e). We examined the senescence of tumor cells, and Figure 6f shows that AX@NPs‐FA/AbCD47 significantly promoted the senescence of tumor cells. In addition, we performed H&E staining of the heart, spleen, lung, and kidney of the mice, and the results showed that the treatments had no toxic effects on these organs (Figure S20, Supporting Information). These findings suggest that AX@NPs‐FA/AbCD47 promoted HCC cellular senescence and inhibited tumor growth by downregulating CD47 and CDC7.

### 2.7 AX@NPs‐FA/AbCD47 Enhanced Antitumor Responses In Vivo

**Figure 7:**
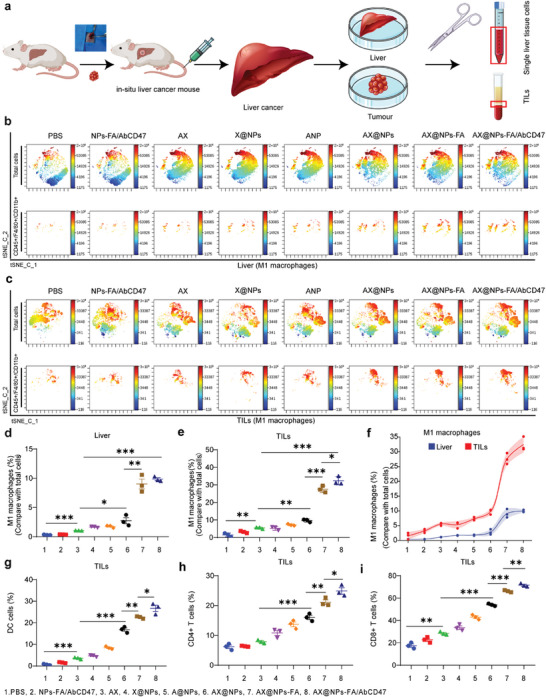
AX@NPs‐FA/AbCD47 increased M1 macrophage differentiation and activated the antitumor immune response. a) Separation of tumor‐infiltrating lymphocytes and single liver tissue cells. b) Flow cytometry analysis of M1 macrophages in hepatocytes. M1 macrophages were identified as CD45+F4/80+CD11b+CD86+. c) Flow cytometry analysis of M1 macrophages among TILs. M1 macrophages were identified as CD45+F4/80+CD11b+CD86+. d) Differences in the percentage of M1 macrophages in adjacent normal hepatocytes after treatment with different drugs. e) Differences in the percentage of M1 macrophages in TILs after treatment with different drugs. f) Differences in the percentage of M1 macrophages in TILs and adjacent normal hepatocytes after treatment with different drugs. g) Statistics of infiltration of mature DCs in TILs of mice treated with different drugs; mature DCs were identified as CD45+CD11c+ CD80+CD86+. h) Statistics of infiltration of CD4+ T cells in TILs of mice treated with different drugs. CD4+ T cells were identified as CD45+CD3+CD4+. i) Statistics of the infiltration of CD8+ T cells in the TILs of mice treated with different drugs. CD8+ T cells were identified as CD45+CD3+CD8+; n = 3, mean ± SEM, ^*^ *p* <0.05, ^**^ *p* <0.01, ^***^ *p* <0.001.

We dissected the livers of C57BL/6 mice at the end of treatment, isolated liver cancer tissues and adjacent liver tissues (>0.5 cm), and extracted tumor‐infiltrating lymphocytes (TILs) and individual liver tissue cells (**Figure** **7**a). Figure 7b–e shows that the AX@NPs‐FA/AbCD47 group exhibited more M1 macrophages (CD45+F4/80+CD11b+CD86+) than the other groups both in liver tissue and among TILs, and the number of M1 macrophages was significantly greater in the tumor tissue than in the adjacent normal liver tissue (Figure 7f). We examined the infiltration of mature DCs and T cells within TILs and found that the numbers of mature DCs, CD4+ helper T cells, and CD8+ cytotoxic T cells were significantly greater after treatment with AX@NPs‐FA/AbCD47 (Figure 7g–i), confirming that AX@NPs‐FA/AbCD47 promoted immune responses and induced immunogenic cell death in tumors. The above results suggest that AX@NPs‐FA/AbCD47 promote antitumor immune responses, especially M1‐type differentiation of macrophages.

## 3 Discussion and Conclusion

Single drugs are prone to off‐target effects and ultimately lead to chemoresistance,^[^ ^48^ ^]^ while dual drugs can solve this problem by preventing drug insensitivity caused by single‐site mutations or the use of alternative pathways.^[^ ^49^ ^]^ Our research revealed that the combined inhibition of CD47 and CDC7 was effective in the treatment of HCC.

To make anti‐CD47 immunotherapy clinically feasible, safety considerations need to be addressed.^[^ ^50^ ^]^ CD47 is highly expressed in HCC,^[^ ^5^ ^]^ but it is also highly expressed in normal erythrocytes,^[^ ^51^ ^]^ and anaemia can occur as a result of the phagocytosis induced by CD47 blockers.^[^ ^52^ ^]^ Currently, the development of CD47 blockers has not been able to overcome the challenge of cytotoxicity (anaemia and hemocytopenia).^[^ ^53^ ^]^ Some organizations have devoted efforts to developing SIRPα fusion proteins, such as TTI‐621 and TTI‐622, that bind only to CD47 on tumor cells but not to CD47 on the surface of erythrocytes.^[^ ^54^ ^]^ However, it is difficult to develop tumor cell‐specific fusion proteins because of the heterogeneity of tumor cells. Researchers have developed bispecific antibodies to CD47 and tumor‐specific antigens to reduce CD47 target‐associated toxicity by binding to other specific targets on the surface of tumor cells while avoiding binding to normal cell, such as HX009, which is a dual targeting agent that targets PD‐1 and CD47.^[^ ^55^ ^]^ This method can effectively improve the targeting of antibody drugs, but antibody drugs are easily cleared in vivo, hindering in situ tumor delivery. To address this problem, we used a dual‐targeting nanodelivery system to deliver an anti‐CD47 drug and a senescence inducer. Nanotechnology was utilized to coload lipid‐soluble and water‐soluble drugs to minimize systemic toxicity, prolong the drug circulation time, and increase drug accumulation within the tumor.^[^ ^56^, ^57^ ^]^ Calcium‐phosphorus NPs are pH‐responsive,^[^ ^58^ ^]^ and our results show that this delivery system hardly targets erythrocytes. The specific mechanism will be investigated in our follow‐up studies.

Our research revealed that the application of NPs for treating HCC precipitates an increase in the number of M1 macrophages among TILs and in the adjacent normal liver tissue. M1 macrophages, which are conventionally linked to anti‐neoplastic responses, secrete cytokines that facilitate the destruction of tumor cells and bolster additional immune mechanisms.^[^ ^59^ ^]^ The elevated numbers of M1 macrophages in TILs signifies an increased immune reaction against tumors.^[^ ^60^ ^]^ NPs possess the capacity to directly incite the maturation or activation of macrophages into the M1 phenotype, which is noted for its antitumor properties. This effect potentially transcends the tumor microenvironment, as evidenced by the increase in M1 macrophages in normal liver tissue, indicating that NP therapy may initiate a systemic immune response extending beyond the localized response restricted to the tumor site. Such systemic activation is advantageous for addressing metastatic cancer cells but concurrently raises concerns regarding potential inflammatory responses or harm to noncancerous tissues. Furthermore, given the pivotal role of the liver in metabolic and detoxification processes, activating immune responses in normal liver tissue warrants cautious evaluation. The possibility of hepatotoxicity or compromised liver functionality, particularly with prolonged nanomedicine treatment or higher dosages, is a relevant risk. Although the upregulation of M1 macrophages through nanomedicinal intervention shows promise in HCC therapy, it also introduces critical considerations regarding systemic immune responses and potential adverse effects. A more comprehensive understanding of its mechanism of action is needed for the development of safer and more efficacious nanomedicine‐based treatments in future endeavors.

In summary, CD47 and CDC7 were found to be highly expressed in patients with HCC, and the clinical data showed that patients with high expression of CD47 and CDC7 had the worst clinical prognosis, suggesting that simultaneously inhibiting CD47 and CDC7 is a promising treatment for HCC. We constructed AX@NPs‐FA/AbCD47, which are dual‐targeting (AbCD47 and FA) NPs that deliver both a CD47 inhibitor and a senescence inducer (a CDC7 inhibitor) to tumor sites. They sequentially release drugs to synergistically augment cellular senescence and immunotherapy and provide novel insight for the clinical treatment of HCC patients.

## 4 Experimental Section

### Cell Culture and Establish the Drug‐Resistant Cells

The human HCC cells (HUH7, HEP3B, Bel‐7402), human normal liver cells (LO2), and mouse HCC cells (Hepa1‐6) were purchased from the Cell Institute of Chinese Academy of Sciences, cultured in DMEM medium containing 10% serum and 1% double antibodies, and placed in a humidified 37 °C and 5% CO2 standing‐temperature incubator. Drug‐resistant Bel‐7402 cells were cultured by incubating them with ascending concentrations of 5‐fluorouracil (Selleck, USA) from 1 to 12 µm for up to 2 months. The drug‐resistant cells were named Bel‐7402R and the parental cells were named Bel‐7402P.

### RNA Sequencing

Shanghai Personalbio Company was commissioned to perform Illumina Novaseq PE150 sequencing of Bel‐7402R and the parental cell line Bel‐7402P. These results were analyzed for Gene Set Enrichment Analysis (GSEA) pathway enrichment analysis, differentially expressed genes, and Kyoto Encyclopedia of Genes, and Genomes (KEGG) pathway analysis.

### Preparation and Characterization of Nanoparticles

DSPE‐PEG2000‐NH_2_, DSPE‐PEG2000‐Cy3, and DSPE‐PEG2000‐FA were purchased from Ponsure Biotechnology (Shanghai, China). N‐(3‐Dimethylaminopropyl)‐N′‐ethylcarbodiimide hydrochloride (EDC), and N‐hydroxysuccinimide (NHS) were purchased from Sigma–Aldrich (St. Louis, USA). AZD8055 and XL413 were obtained from Selleck (Shanghai, China), and other relative reagents were obtained from Sigma–Aldrich (USA). AZD8055 and XL413 were encapsulated with DSPE‐PEG2000‐NH_2_ or DSPE‐PEG2000‐FA by thin film hydration; where AZD8055 was encapsulated on the inner side, while XL413 was adsorbed on the nanomaterial surface. Subsequently, CaCl_2_ solution and HBS (Hepes, Na_3_PO_4_, and NaCl; pH 7.4) buffer were added to adsorb calcium and phosphorus onto the nanoparticles. AX@NPs or AX@NPs‐FA was formed by standing for 30 min at room temperature. The surface of the NPs was then modified by the standard EDC/NHS‐mediated amide reaction with ultra‐LEAF purified anti‐mouse or anti‐human CD47 blocking antibody (AbCD47) according to previous reports and the previous protocols.^[^ ^61^, ^62^ ^]^ Briefly, AbCD47 (100 µg mL^−1^), EDC (0.1 m), and NHS (0.1 m) were added to NPs‐FA solution (5 mg mL^−1^) and gently stirred for 2 h. The resulting NPs‐FA/AbCD47 solution was centrifuged at 15 000 g for 15 min and the supernatant was discarded to remove unbound antibody. The NPs‐FA/AbCD47 solution was stored at 4 °C. Fourier Transform Infrared Spectrometer (FTIR) (NEXUS 670, Nicolet, USA) detection of the conjugation of AbCD47 and NPs‐FA; The 12.5% SDS‐PAGE gel (Epizyme, China) was prepared, and 4 ug of free AbCD47 and the same dose of NPs‐FA/AbCD47 were added for electrophoresis, stained with coomassie blue fast staining (Epizyme, China) for 15 min, and subsequently photographed for analysis the conjugation of AbCD47 and NPs‐FA; Zetasizer IV analyzer (Malvern Zetasizer Nano ZS90, Malvern, U.K.) to detect the particle size and potential of nanoparticles; Transmission electron microscope (TEM) (FEI Tecnai G2 Spirit TEM, USA) to examine the morphology of nanoparticles; Shapes of NPs‐FA/AbCD47 observed by atomic force microscopy (AFM) (Hatachi, Japan); Calcium and phosphorus elemental analysis of NPs was performed using field emission TEM (Talos F200X, USA). The high‐performance liquid chromatography (HPLC) (Agilent 1100, USA) was used to measure the encapsulation efficiency (EE%) of AZD8055 and XL413 in AX@NPs‐FA/AbCD47. To carry out some other experiments, Dir (Biotium, USA) or rhodamine B (RB) (Beyotime, China) were applied to prepare Dir@NPs and Dir@NPs‐FA and Dir@NPs‐FA/AbCD47 or RB@NPs and RB@NPs‐FA and RB@NPs‐FA/AbCD47, respectively.

### Erythrocyte Targeting Assay

Hepa1‐6 were inoculated into 1.5 cm glass‐bottomed culture dishes overnight at 5000 cells well^−1^. Next day, C57BL/6 mice were subjected to orbital blood sampling to extract erythrocytes. 1 × 10^5^ mouse erythrocytes were added to the dishes inoculated with Hepa1‐6 in advance. RB@NPs‐FA/AbCD47 were added and incubated for 4 h. Photographs were taken with a laser confocal microscope (Olympus FV1000, Japan).

### In Vitro AZD8055 and XL413 Release from Nanoparticles

A dialysis assay was applied to confirm the release profiles of AZD8055 and XL413 from the nanoparticles in vitro. The nanoparticles were dialyzed against a PBS solution containing 10 m sodium salicylate, adjusted to either pH 6.0 or pH 7.4. At predetermined time points, 100 µL of the release solution was collected for subsequent analysis, and an equal volume of fresh dialysate was added. The analysis was performed with an Agilent 2000 HPLC instrument equipped with a C18 column. The mobile phase comprised potassium dihydrogen phosphate (KH_2_PO_4_) at pH 4.6, acetonitrile, and methanol. The flow rate was maintained at 1 mL min^−1^, and the injection volume was set at 10 µL. UV detection was performed at a wavelength of 254 nm.

### Cytotoxicity Assay

Cells were inoculated into 96‐well plates at 3000 cells/well and left overnight, then treated with drug and incubated for 72 h. The medium was aspirated off, medium containing CCK8 (Beyotime, China) was added, incubated for 1 h, and the OD value at 450 nm was detected with an enzyme marker (Thermo MultisKan FC, USA).

### Western Blotting Assay

Cells were seeded in 6‐well plates at a density of 3 × 10^5^ cells per well and incubated overnight. Subsequently, the cells were treated with drugs for 48 h. Following treatment, the culture medium was discarded, and the cells were washed with PBS. Cell lysis was achieved by the addition of RIPA buffer, supplemented with both phosphatase and protease inhibitors (both from Beyotime, China). The cell lysates were then collected into tubes to facilitate protein extraction and the measurement of protein concentrations (Beyotime, China). Protein electrophoresis was conducted using 12% SDS polyacrylamide gel electrophoresis (PAGE) gels (Beyotime, China), after which the separated proteins were transferred onto polyvinylidene difluoride (PVDF) membranes. The membranes were subsequently blocked with 10% (w/v) nonfat milk powder for 1 h. Overnight incubation at 4 °C with primary antibodies was followed by a 1 h incubation with appropriate horseradish peroxidase‐conjugated secondary antibodies (mouse or rabbit, from Proteintech, USA) the following day. Detection was performed using an enhanced chemiluminescence (ECL) solution from Sigma (USA). Antibodies targeting CD47 and GAPDH were acquired from Abcam (USA).

### Drug Synergy Experiment

Cells were inoculated into 96‐well plates at 3000 cells well^−1^ and left overnight. The next day, the drug was added for 72 h. The OD values were measured according to the cytotoxicity assay described above and the IC50 values were calculated using GraphPad Prism 8.0. The Chou‐Talalay method (CI <1 indicates synergism) was applied to detect the synergism through using the CompuSyn software (Ver.1.0).

### Apoptosis Assay

Cells were inoculated into 6‐well plates at 3×10^5^ cells well^−1^ left overnight, and treatment with drugs for 48 h the next day. Cells were collected and stained with Annexin V‐PI apoptosis detection kit (Vazyme, China) and assayed using flow cytometry (Becton Dickinson, USA). Data were analyzed and processed with FlowJo 10. Each experiment was repeated three times.

### SA‐β‐Gal Staining

SA‐β‐Gal staining was performed in 6 wells (for in vitro studies) or on 10 µm thick frozen sections of tumor tissue (for in vivo studies) using the β‐Galactosidase staining kit (Beyotime, China), according to the manufacturer's instructions.

### LDH Detection Experiment

Cells were inoculated into 24‐well plates at 5 × 10^5^ cells well^−1^ overnight and dosed for 48 h. The plates were centrifuged at 400 g for 5 min using a multi‐well plate centrifuge. 120 µL of supernatant from each well was carefully aspirated into a 96‐well plate and subsequently assayed according to the instructions of the Lactate Dehydrogenase Cytotoxicity Assay Kit (Beyotime, China). The measured data were analyzed and processed with GraphPad Prism 8.0. Each experiment was repeated three times.

### ROS Detection Experiment

Cells were inoculated into 24‐well plates at 5 × 10^5^ cells well^−1^ and left overnight, and drugged for 48 h. Staining was performed according to the instructions of the reactive oxygen species detection kit (Beyotime, China), and subsequent photographic analysis was performed with a fluorescence microscope (Olympus Corporation, Japan).

### In Vitro Phagocytosis Assay

The assay was performed as described previously.^[^ ^63^ ^]^ THP‐1 cells were inoculated into 12‐well plates at 1 × 10^5^ cells well^−1^, while Phorbol‐12‐myristate‐13‐acetate (PMA) (Sigma, USA) was added for 48 h to induce THP‐1 into walled macrophages. After macrophages were treated with serum‐free culture starvation for 2 h, 1 × 10^5^ hepatocellular carcinoma cells (HEP3B and HUH7) stained in advance with Calcium‐AM were added and then co‐cultured in complete medium containing drugs for 2 h. The medium was aspirated and washed three times with PBS to remove free cells. Photographs were taken with a fluorescent microscope (Olympus Corporation, Japan). Employing the phagocytic index—a metric denoting the number of HCC cells ingested by macrophages for every 100 macrophages—the phagocytosis of HCC cells were quantified by macrophages following a variety of drug treatments.

### Cellular Uptake Assay

Cells were inoculated into 6‐well plates overnight at 1×10^5^ cells well^−1^ and added free RB (Beyotime, China) or RB@NPs or RB@NPs‐FA or RB@NPs‐FA/AbCD47 for 1 and 4 h, respectively, and then two experiments were performed as follows: 1) fixed with 4% paraformaldehyde and stained with DAPI (Beyotime, China) for 10 min and photographed with a laser confocal microscope (Zeiss LSM510, Germany); 2) live cells were collected and assayed using flow cytometry (Becton Dickinson, USA), and the data were analyzed and processed with FlowJo 10. Each experiment was repeated three times.

### Lysosomal Escape Assay

Cells were inoculated into 1.5 cm glass bottom dishes overnight at 1 × 10^5^ cells well^−1^, and RB@NPs‐FA/AbCD47 was added for 1 and 4 h, respectively. The green LysoTracker probe (Beyotime, China) was added for 40 min. Photographs were taken with a laser confocal microscope (Olympus FV1000, Japan).

### In Vitro Mitochondrial Targeting Assay

Cells were inoculated into 1.5 cm glass bottom dishes overnight at 1×10^5^ cells well^−1^, and NPs‐Cy3‐FA/AbCD47 was added for 1 h and 4 h, respectively, after which the medium was replaced with fresh medium. Then Mito‐Tracker Green (Beyotime, China) was added to stain for 30 min and Hoechst 33 342 was added for 10 min. Photographs were taken with a laser confocal microscope (Olympus FV1000, Japan).

### Immunofluorescence Assay

Cells were inoculated into 6‐well plates overnight at 1 × 10^5^ cells well^−1^, treated with or without drugs the next day, fixed with 4% paraformaldehyde for 10 min, broken with Triton‐100, blocked with 5% BSA, incubated overnight at 4 °C with specific primary antibody, incubated for 3 h with the corresponding fluorescent secondary antibody, the nuclei were stained with DAPI (Beyotime, China), and photographed using a laser confocal microscope (Zeiss LSM510, Germany). The primary antibodies used were CD47 (abcam, USA) and CDC7 (abcam, USA). The fluorescent secondary antibodies used were Alexa Fluor 488‐labeled goat anti‐rabbit IgG (Beyotime, China) and Alexa Fluor 647‐labeled goat anti‐mouse IgG (Beyotime, China).

### Animal Surgeries

All procedures in this study were performed in accordance with the guidelines established by the Institutional Animal Care and Use Committee at Renji Hospital, School of Medicine, Shanghai Jiao Tong University (Shanghai, China). All surgeries were performed using aseptic conditions.

### The Tumor Targeting Abilities of NPs In Vivo

To encapsulate Dir to form Dir@NPs, Dir@NPs‐FA, and Dir@NPs‐FA/AbCD47 nanomaterials were used. In advance, HUH7 tumor cells were inoculated over the root of the right thigh of mice at the number of 2×10^6^ cells, and when the tumors grew to 200–300 mm^3^, the mice were intravenously administered 100 µL of Dir@NPs or Dir@NPs‐FA or Dir@NPs‐FA/AbCD47 at a 0.5 µg/mL Dir dose. Since the lipophilic tracer Dir had a strong fluorescence emission in the near‐infrared region (λex/λem: 748/780 nm), it could be used to detect the biodistribution of NPs in tumor‐bearing nude mice. After injection, whole‐body fluorescence images were obtained at 8, 24, 48, and 72 h time points using an in vivo imaging system (Berthold Technologies, Germany). Mice were sacrificed and dissected 72 h after injection, to examine the distribution of NP in the major organs (spleen, heart, liver, lung, and kidney).

### Anticancer Activity In Vivo

Three‐week‐old BALB/c nude male mice and C57BL/6 male mice were obtained from the Animal Technology Co., Ltd. (Beijing, China). Hepa1‐6 and HUH7 tumor cells were inoculated at 2 × 10^6^ cell volume above the root of the right thigh of mice, respectively. All procedures in this study were performed in accordance with the guidelines established by the Institutional Animal Care and Use Committee at Renji Hospital, School of Medicine, Shanghai Jiao Tong University (Shanghai, China). All surgeries were performed using aseptic conditions. When the tumors grew to ≈900 mm^3^, the mice were executed and dissected, the tumors were cut into 1.5 mm × 1.5 mm × 1.5 mm tubercles, the mice were anesthetized with an inhalation anesthesia machine (isoflurane), sterilized, and the abdomen was opened to expose the liver, the tubercles were inoculated into the liver with forceps, and the mice were sterilized and sutured. The mice were treated from the 7th postoperative day. The treatment strategies for HUH7 tumor‐bearing nude mice (n = 5 per group): 1) PBS, 2) NPs‐FA/AbCD47, 3) AZD8055 (5 mg kg^−1^), 4) XL413 (25 mg kg^−1^), 5) AZD8055+XL413(5 and 25 mg kg^−1^), 6) X@NPs at a XL413‐equivalent dosage of 25 mg kg^−1^, 7) A@NPs at a AZD8055‐equivalent dosage of 5 mg kg^−1^, 8) AX@NPs at a AZD8055‐equivalent dosage of 5 mg kg^−1^ and XL413‐equivalent dosage of 25 mg kg^−1^, 9) AX@NPs‐FA at a AZD8055‐equivalent dosage of 5 mg kg^−1^ and XL413‐equivalent dosage of 25 mg kg^−1^, 10) AX@NPs‐FA/AbCD47 at a AZD8055‐equivalent dosage of 5 mg kg^−1^ and XL413‐equivalent dosage of 25 mg kg^−1^; The treatment strategies for Hepa1‐6 tumor‐bearing C57BL/6 mice (n = 5 per group): 1) PBS, 2) NPs‐FA/AbCD47, 3) AZD8055+XL413(5 and 25 mg kg^−1^), 4) X@NPs at a XL413‐equivalent dosage of 25 mg kg^−1^, 5) A@NPs at a AZD8055‐equivalent dosage of 5 mg kg^−1^, 6) AX@NPs at a AZD8055‐equivalent dosage of 5 mg kg^−1^ and XL413‐equivalent dosage of 25 mg kg^−1^, 7) AX@NPs‐FA at a AZD8055‐equivalent dosage of 5 mg kg^−1^ and XL413‐equivalent dosage of 25 mg kg^−1^, 8) AX@NPs‐FA/AbCD47 at a AZD8055‐equivalent dosage of 5 mg kg^−1^ and XL413‐equivalent dosage of 25 mg kg^−1^. The drug was administered by tail vein injection, twice a week for a total of 3 weeks, and the body weight of the mice was recorded at each treatment. Tumor growth in nude mice was recorded weekly by small animal ultrasound. The tumor growth inhibition (TGI) value was calculated as follows: TGI = 100% × (1 – treatment group/control group). The mice were sacrificed, and preserve the tumors and vital organs (heart, liver, spleen, lung, and kidney) of C57BL/6 mice. Vital organs were fixed with 4% paraformaldehyde and stained using hematoxylin‐eosin (H&E) staining. The cleaned tumors were weighed and fixed with 4% paraformaldehyde and then sectioned. Immunohistochemistry (IHC) was performed using standard methods. The sections were stained with TUNEL, CD31, CD47, CDC7, and mTOR antibodies. The CD47, CDC7, and mTOR antibodies were purchased from Abcam (USA), TUNEL and CD31 antibodies were purchased from Beyotime (China). The major organs were also fixed, sectioned, and examined by H&E staining.

### Tissue Dissociation and Flow Cytometry

C57BL/6 mice were executed with CO_2_ asphyxiation apparatus. All tumor tissues and liver tissues were divided into 2 parts for subsequent flow cytometry and histopathology preparation. Tumors and liver tissues were immersed in pre‐cooled PBS, cut with scissors, wash, and then added to DMEM containing 2.0 mg mL^−1^ Collagenase IV (Sigma, USA) and 50 units/mL DNase I (Roche, Basel, Switzerland). The cells were digested for 1 h at 37 °C and filtered through 70‐µm cell filter (Biosharp, China) to make single‐cell suspensions. Tumor cells were added to percoll (GE, USA) to extract tumor‐infiltrating lymphocytes (TILs). RBC Lysis Buffer (BioLegend, USA) was added to the TILs and hepatocytes for 10 min at room temperature, centrifuged and resuspended. Subsequent staining with FVS780 (BD, USA) at room temperature for 15 min to differentiate between dead and live cells; Cells were then blocked with an Fc Receptor Binding Inhibitor (eBioscience, USA) for 10 min at room temperature. Staining the cells with fluorescently labeled primary monoclonal antibodies for 30 min. The following antibodies were used for analysis of TILs and liver macrophages: anti‐CD11b‐FITC, anti‐F4/80‐Brilliant Violet 421, anti‐CD86‐Brilliant Violet 605, anti‐CD206‐Brilliant Violet 650, anti‐CD45‐PE, Fixable Viability Stain 780 (BD, USA) stain was added to exclude dead cells; The following antibodies were used for analysis of TILs DC cells: anti‐CD11c‐APC, anti‐CD86‐Brilliant Violet 605, anti‐CD80‐FITC, anti‐CD45‐PE, Fixable Viability Stain 780 (BD, USA) stain was added to exclude dead cells; The following antibodies were used for analysis of TILs T cells: anti‐CD3‐Brilliant Violet 421, anti‐CD4‐Brilliant Violet 605, anti‐CD8‐Brilliant Violet 650, anti‐CD45‐PE, Fixable Viability Stain 780 (BD, USA) stain was added to exclude dead cells. Acquisition was performed on the flow cytometry (Becton Dickinson, USA). Data analysis was performed using FlowJo Version 10.8.1. TILs and liver M1‐macrophages were identified as CD45+CD11b+F4/80+CD86+; TILs DC cells were identified as CD45+CD11c+CD80+CD86+; TILs CD4+T cells were identified as CD45+CD3+CD4+; TILs CD8+T cells were identified as CD45+CD3+CD8+.

### In Vivo Drug Release and Mitochondrial Targeting Assay

The Hepa1‐6 cells were injected into the above the root of the right thigh of three‐week‐old BALB/c nude male mice at 2 × 10^6^, and when the tumors grew to 800 mm^3^, the tail vein was administered with the nanodrug Calcein/RB@NPs‐FA/AbCD47 which calcein was adsorbed on the outer side of nanoparticles and rhodamine (RB) was wrapped on the inner side, and the animals were photographed at 0.5 and 6 h using a chemiluminescent fluorescence image analysis system (PerkinElmer, USA). Then, the mice were sacrificed, and preserve the tumors. Tumor tissues were prepared as electron microscopy samples and photographed using TEM.

### Drug‐Resistant Liver Cancer Treatment in Mice and Photoacoustic Imaging

Bel‐7402R cells (2 × 10^6^) were inoculated above the root of the right thigh of three‐week‐old BALB/c nude male mice, and the therapy was started when they grew to ≈80 mm^3^.The treatment strategies for Bel‐7402R tumor‐bearing nude mice (n = 5 per group): (1) PBS, (2) AX@NPs‐FA/AbCD47 at a AZD8055‐equivalent dosage of 5 mg kg^−1^ and XL413‐equivalent dosage of 25 mg kg^−1^. The drug was administered by tail vein injection, twice a week for a total of 4 weeks. Photoacoustic imaging of small animals (Vevo, Japan) was used to record the intratumoral blood oxygenation of the animals and measure the values. Then, the mice were sacrificed, and preserve the tumors. The cleaned tumors were weighed and fixed with 4% paraformaldehyde. Immunohistochemistry (IHC) was performed using standard methods. The sections were stained with CD47, CDC7, mTOR, and H2AX antibodies. The CD47, CDC7, and mTOR antibodies were purchased from Abcam (USA), and H2AX antibody was purchased from Sigma (USA).

### TCGA Database Analysis

The gene expression of CD47, CDC7, and FOLR1 were downloaded from TCGA database and the corresponding information of liver cancer patients, divided the liver cancer patients into two groups with high and low expression according to the expression of each gene, and analyzed the survival time of the two groups with GraphPad Prism 8.0 software.

### TIMER Database Analysis

The correlation between CD47 and CDC7 in HCC was analyzed by running the TIMER database (http://timer.comp‐genomics.org/).

### Molecular Docking

The PDB file of the CD47 crystal structure (PDB code: 2VSC) was downloaded from Protein Bank (www.pdb.org). The 3D structure of AZD8055 was modeled by Schrodinger, and AZD8055 was docked to CD47 via using the standard precision scoring function of Glide 5.5 software (Schrödinger, USA). The heterozygous site formed by residues Lys39 and Tyr37 was finally selected to define and generate the receptor grid.

### Statistical Analysis

GraphPad Prism 8.0 software was used in this study to perform statistical analysis of the experimental data. The analysis of data across two distinct groups was performed utilizing the Student's t‐test, when appropriate for the dataset. For comparisons involving multiple groups, analysis of variance (ANOVA) was used to determine the statistical significance of differences. Survival analyses were conducted using the Kaplan‐Meier method, with subsequent comparisons made through the log‐rank test. The data were represented as mean ± standard errors of the mean (SEM). Statistical significance was established at a p‐value threshold of ≤0.05. The symbols ^*^ *p* ≤0.05, ^**^ *p* ≤0.01, and ^***^ *p* ≤0.001 indicate levels of significance, whereas a p‐value ≥ 0.05 was considered to indicate that the difference was not significant, denoted as ns. Unless otherwise stated, each experiment was repeated at least three times.

## Conflict of Interest

The authors declare no conflict of interest.

## Author Contributions

K.G., J.Y.J., and Z.H.W. contribute equally to this work. K.G., J.Y.J., Z.H.W., Q.W., J.H.L., Y.D., and J.T.L. conducted experiments. J.Y.J. conducted in situ implant surgery. K.G., J.Y.J., and Z.H.W. analyzed the data. J.Y. and Y.S. provided materials and assistance. K.G., Y.Z., and Y.R.D wrote the paper, with input from all authors. Y.R.D supervised the study.

## Supporting information

Supporting Information

## Data Availability

The data that support the findings of this study are available from the corresponding author upon reasonable request.
