# Supplementary material for: Nanosystem Delivers Senescence Activators and Immunomodulators to Combat Liver Cancer
Source: Adv Sci (Weinh). 2024 Mar 23;11(20):2308310. doi: 10.1002/advs.202308310 (PMC11132057; doi:10.1002/advs.202308310)
Supplement: Supplementary file 1 — Supporting Information [file ADVS-11-2308310-s001.pdf]

ADVANCED SCIENCE
Open Access

Supporting Information

for *Adv. Sci.*, DOI 10.1002/advs.202308310

Nanosystem Delivers Senescence Activators and Immunomodulators to Combat Liver Cancer

Ke Gong, Juyang Jiao, Zhihua Wu, Quan Wang, Jinghan Liao, Yi Duan, Jiangtao Lin, Jian Yu, Ying Sun, Yong Zhang and Yourong Duan**

Supporting Information

Nanosystem Delivers Senescence Activators and Immunomodulators to Combat Liver Cancer

Ke Gong[#], Juyang Jiao[#], Zhihua Wu[#], Quan Wang, Jinghan Liao, Yi Duan, Jiangtao Lin, Jian Yu, Ying Sun, Yong Zhang[*], and Yourong Duan[*]

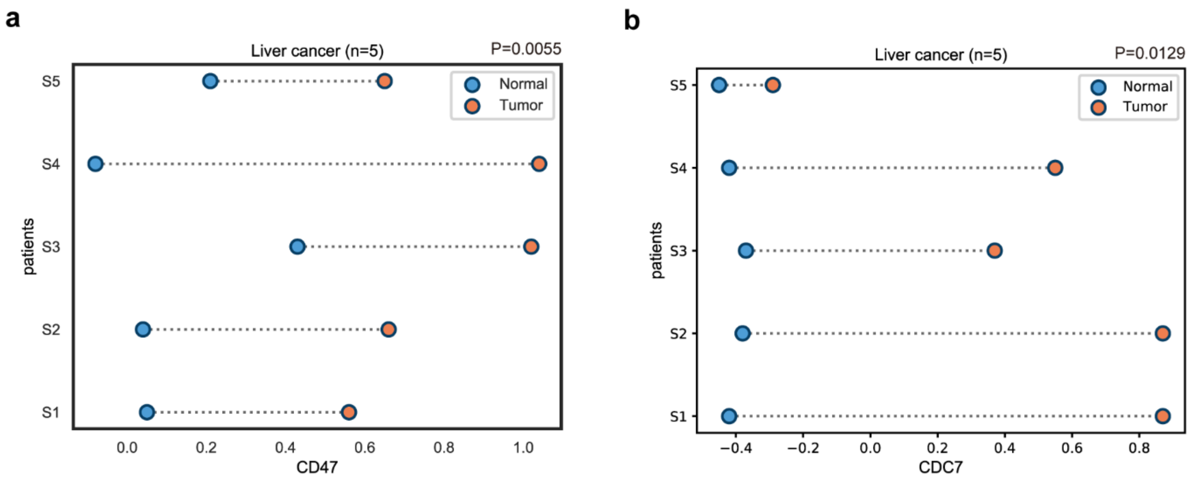

Figure S1. High expression of CD47 and CDC7 was detected in 5 patients with HCC. **a.** Analysis of CD47 expression in 5 liver cancer patients (tumour tissue and adjacent normal tissue) in GSE146049. **b.** Analysis of CDC7 expression in 5 liver cancer patients (tumour tissue and adjacent normal tissue) in GSE146049. A p value less than 0.05 was considered to indicate statistical significance.

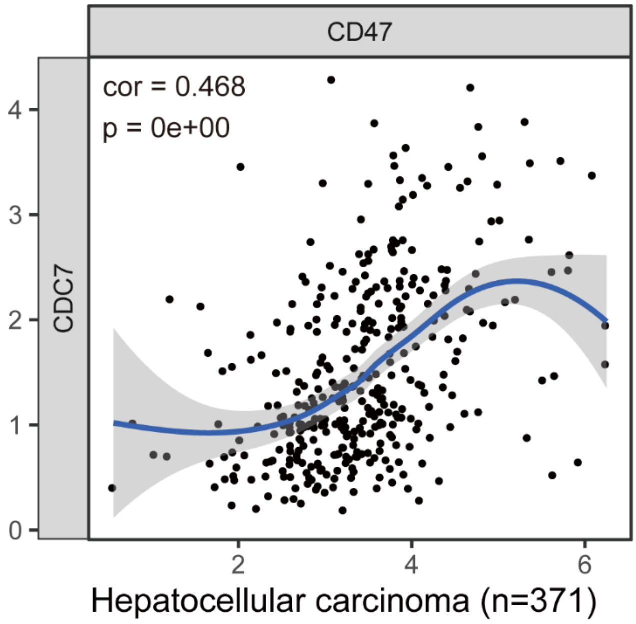

Figure S2. The TIMER database was used to analyse the relationship between CD47 and CDC7 in hepatocellular carcinoma (371 samples).

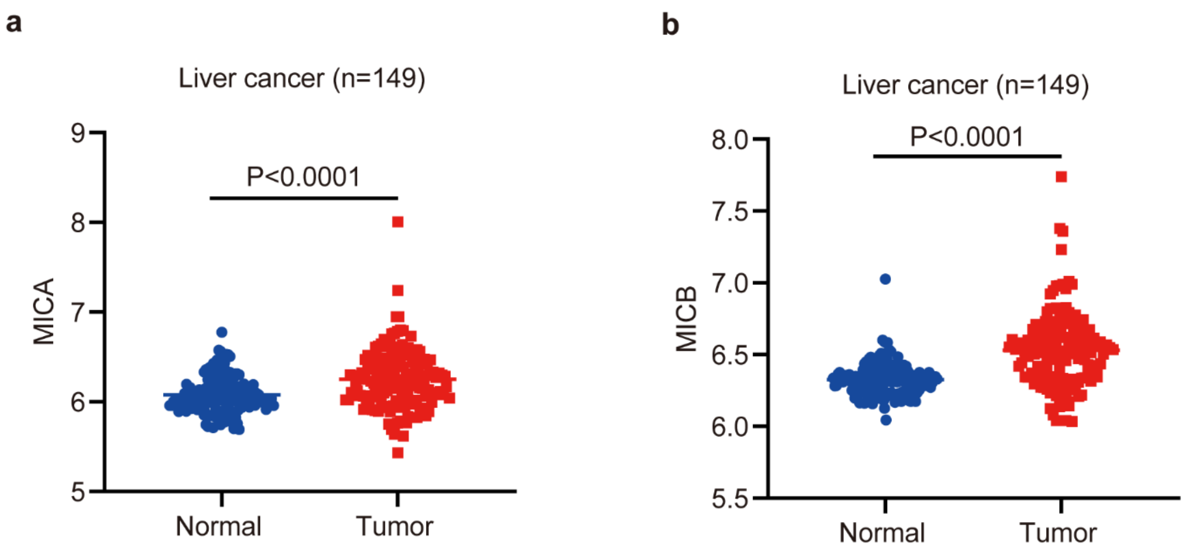

Figure S3. The expression of the MHC class I molecules MICA (**a**) and MICB (**b**) was analysed in 149 samples of HCC tissues and adjacent normal tissues, a p value less than 0.05 was considered to indicate statistical significance.

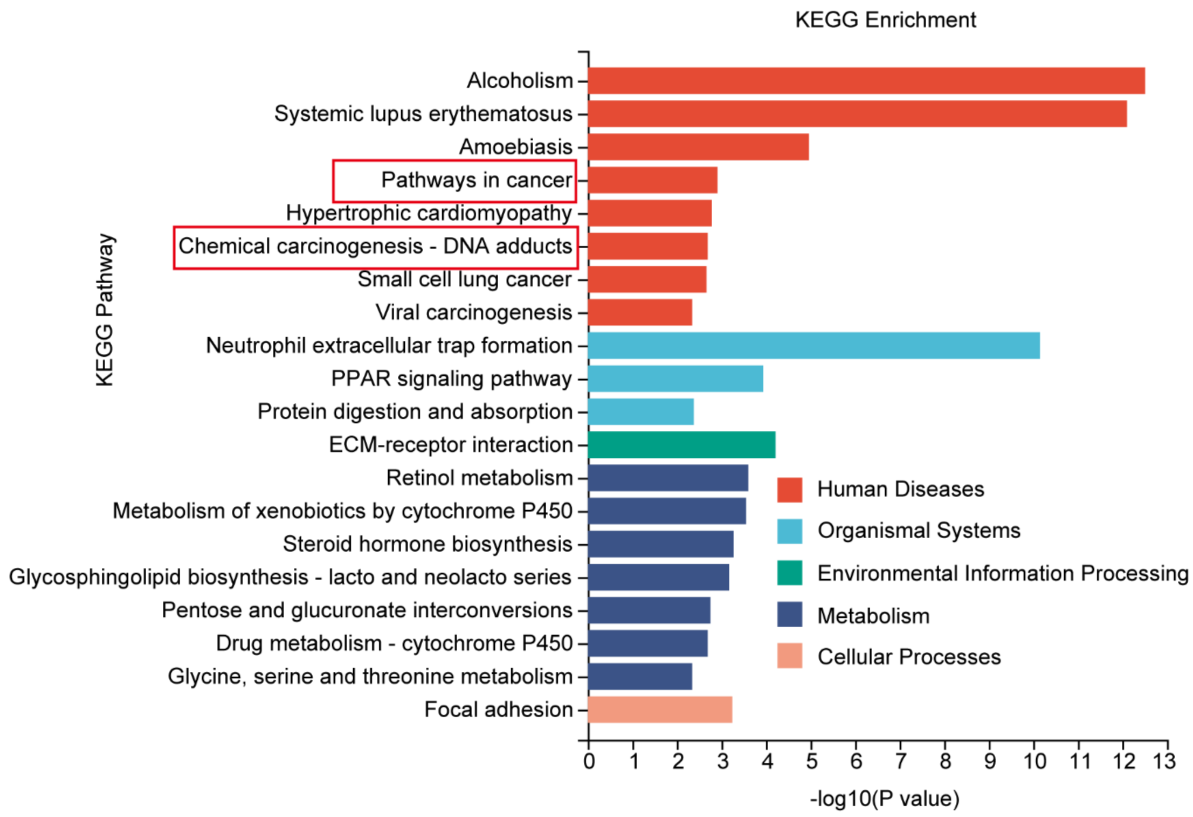

Figure S4. KEGG pathway analysis of Bel-7402P and Bel-7402R cells.

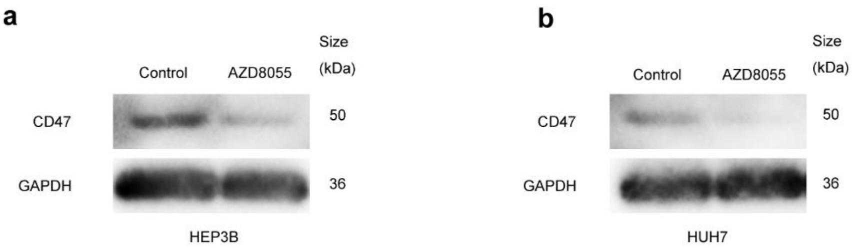

Figure S5. The Western blot results suggest that AZD8055 downregulates CD47 expression in both HEP3B (**a**) and HUH7 (**b**) cell lines.

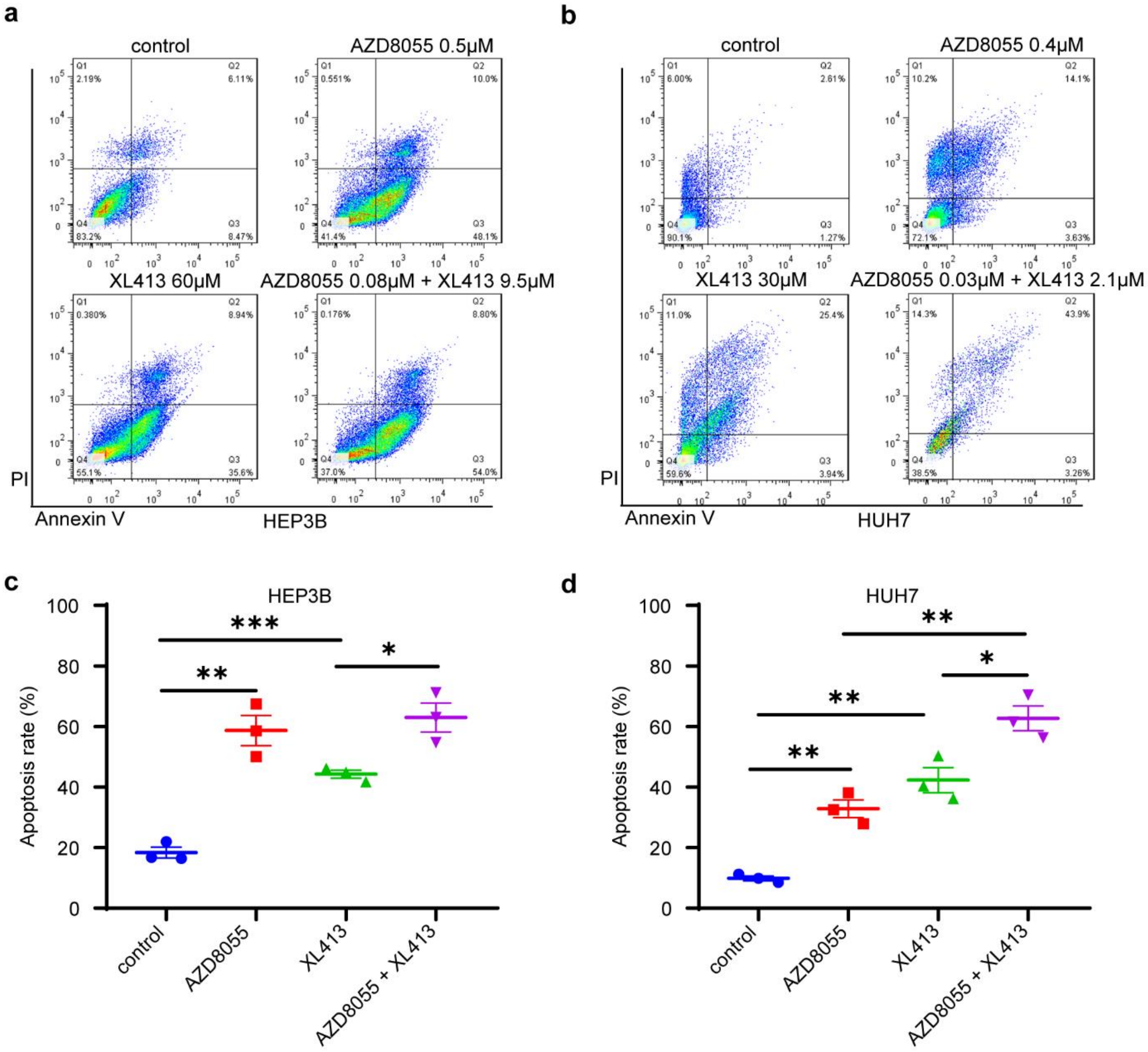

Figure S6. a-b. Flow cytometry was used to evaluate the apoptosis of HEP3B and HUH7 cells after treatment with drugs (HEP3B: control, 0.5 μM AZD8055, 60 μM XL413, 0.08 μM AZD8055 + 9.5 μM XL413; HUH7: control, 0.4 μM AZD8055, 30 μM XL413, 0.03 μM AZD8055 + 2.1 μM XL413). **c-d.** Statistical analysis of the apoptosis rates of HEP3B and HUH7 cells. n=3, mean ± SEM, *$p < 0.05$, **$p < 0.01$, ***$p < 0.001$.

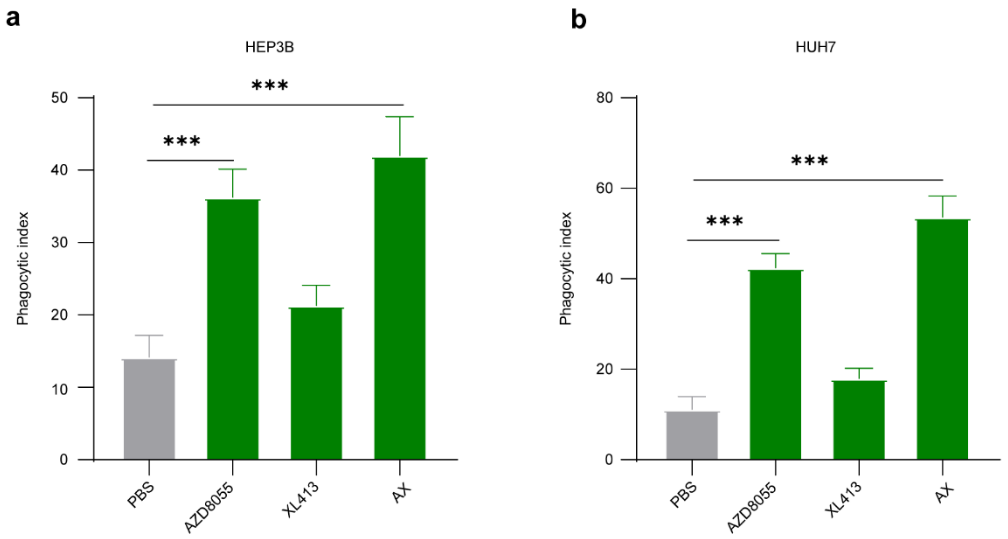

Figure S7. Statistical evaluation was performed to assess the phagocytosis of HEP3B (**a**) and HUH7 (**b**) cells by macrophages after the administration of AZD8055, XL413, or a combination of AZD8055 and XL413. n=5, mean ± SEM, ***P < 0.001.

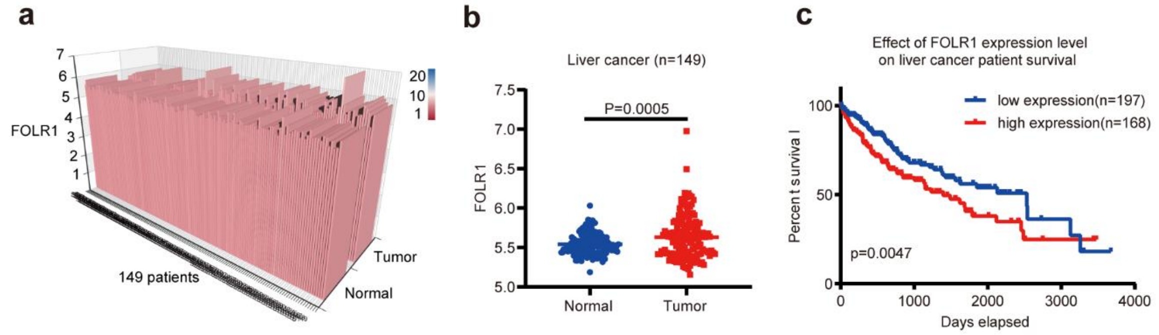

Figure S8. FOLR1 was highly expressed in patients with HCC and was associated with a poor prognosis. **a.** The expression of FOLR1 in 149 patients with HCC. **b.** Statistical analysis of FOLR1 expression in 149 patients with HCC. **c.** TCGA database analysis of the associations between FOLR1 expression and prognosis in patients with HCC. A p value less than 0.05 was considered to indicate statistical significance.

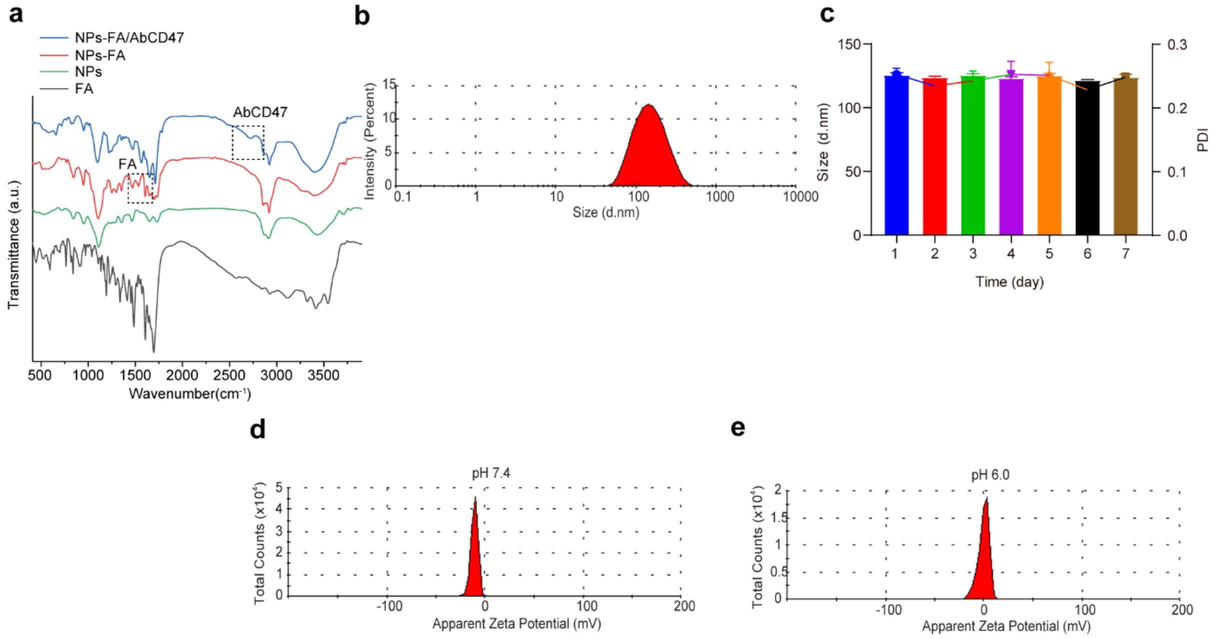

Figure S9. Characterization of NPs. **a.** Detection of FA, NPs, NPs-FA and NPs-FA/AbCD47 by near-infrared spectroscopy. **b.** Size of NPs. **c.** Changes in the size and PDI of NPs incubated in a solution containing 5% FBS for 7 days. **d.** Zeta potential of NPs at pH 7.4. **e.** Zeta potential of NPs at pH 6.0.

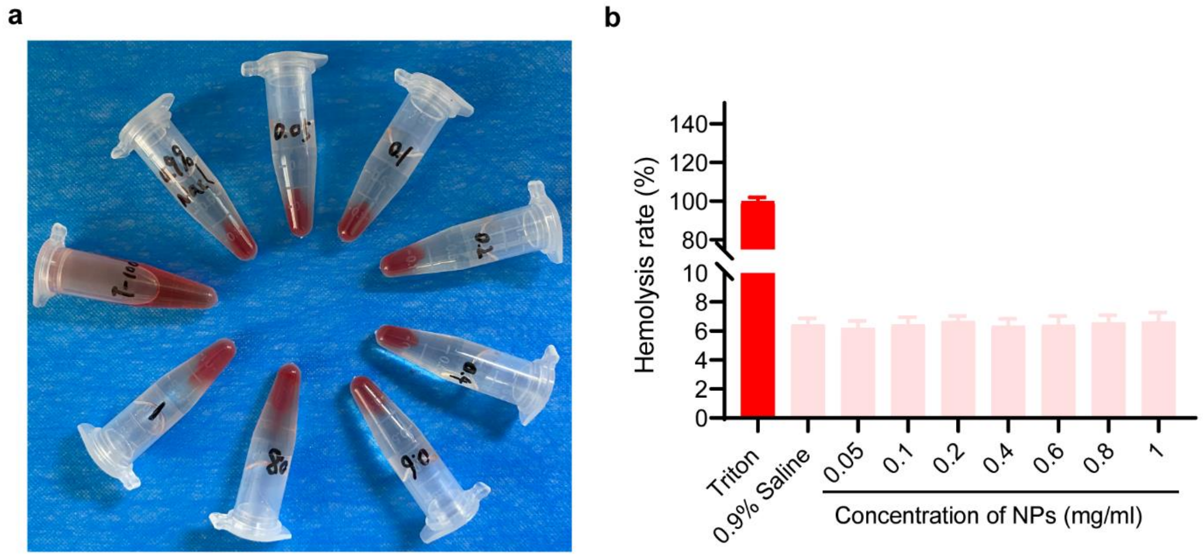

Figure S10. The biological safety of NPs. **a.** A haemolysis assay with various concentrations of NPs was performed with Triton X-100 as a positive control and 0.9% saline as a negative control. **b.** Statistical results of the haemolysis assay.

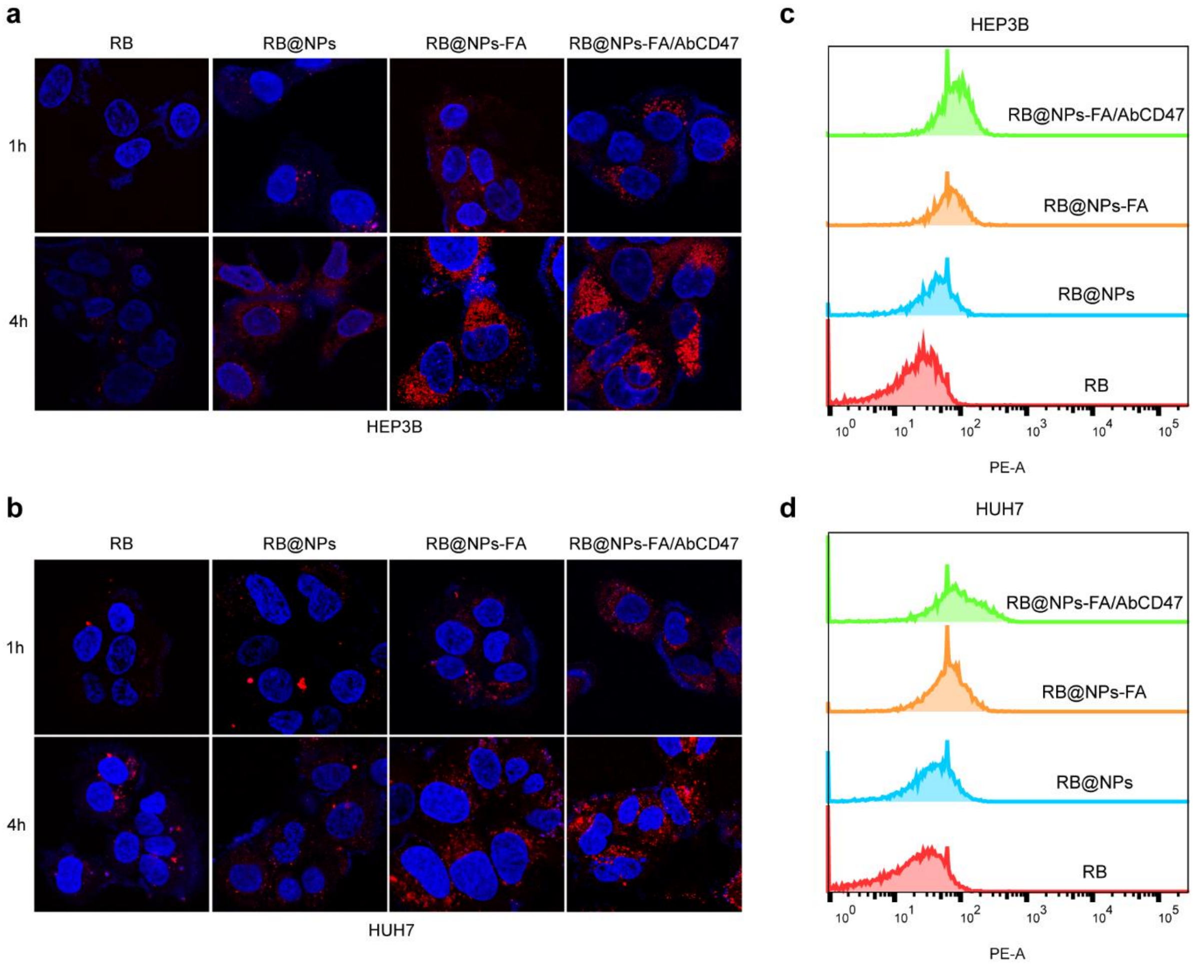

Figure S11. a-b. HEP3B and HUH7 cells were incubated with free RB, RB@NPs, RB@NPs-FA and RB@NPs-FA/AbCD47 for 1 h and 4 h, and drug uptake by liver cancer cells was observed with a fluorescence microscope. Red represents RB or NP-encapsulated RB; blue represents the nucleus. **c-d.** HEP3B and HUH7 cells were incubated with free RB, RB@NPs, RB@NPs-FA, or RB@NPs-FA/AbCD47 for 4 h, and drug uptake by liver cancer cells was evaluated by flow cytometry.

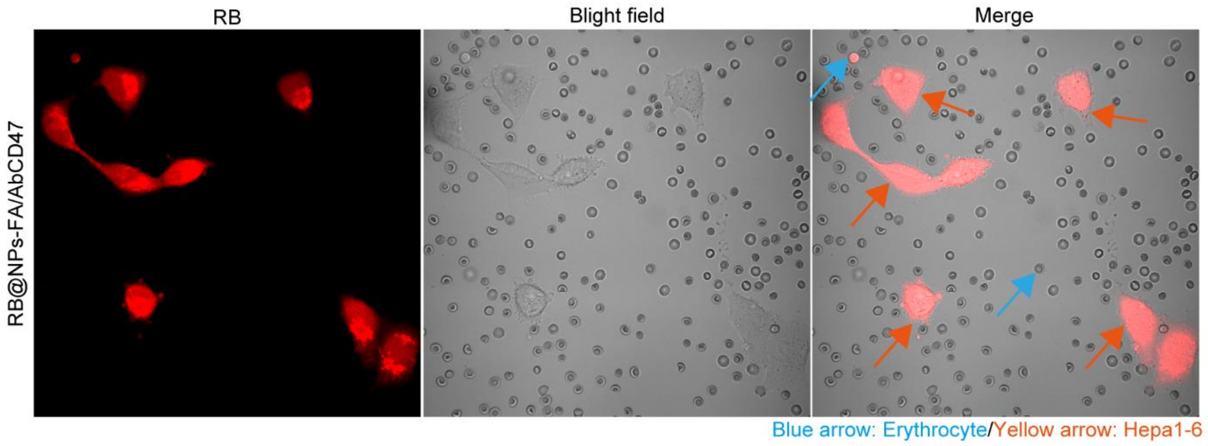

Figure S12. RB@NPs-FA/AbCD47 were added to dishes coincubated with erythrocytes and Hepa1-6 cells for 4 h. Blue arrow indicates erythrocytes, and yellow arrow indicates Hepa1-6 cells.

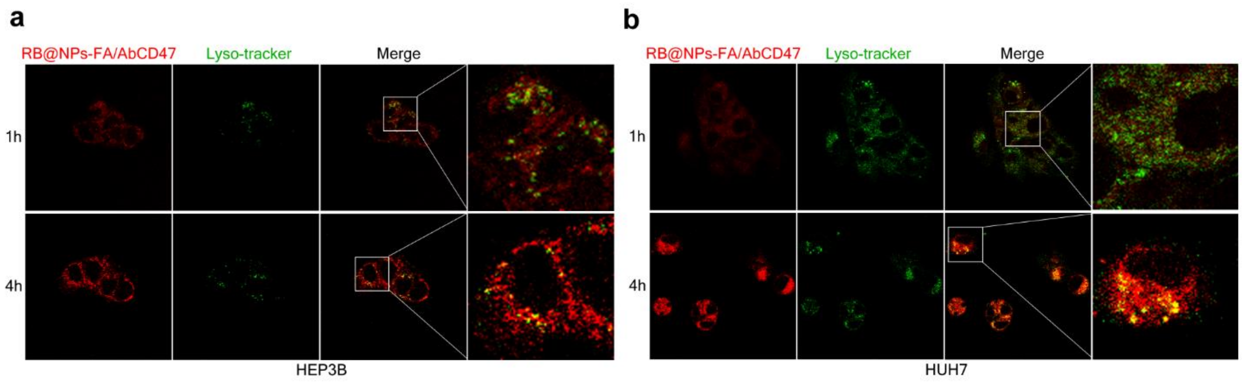

Figure S13. a-b. HEP3B and HUH7 cells were incubated with RB@NPs-FA/AbCD47 for 1 h and 4 h. The escape of NP-encapsulated RB from lysosomes was monitored by fluorescence microscopy. Red represents NP-encapsulated RB; green represents lysosomes. Red and green show that the NP is not phagocytosed by lysosomes; red and green superimposed indicate that the NP is phagocytosed by lysosomes.

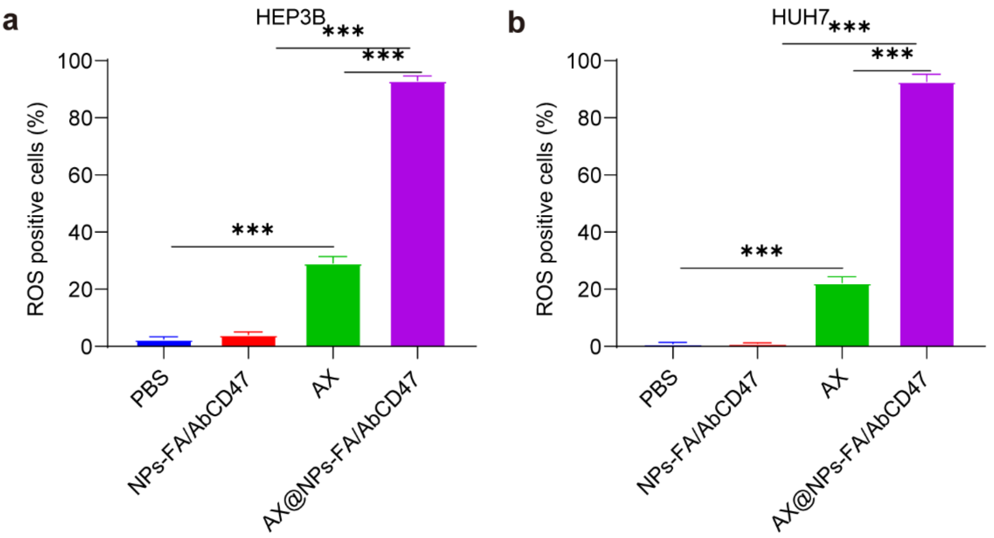

Figure S14. a-b. Statistics of intracellular ROS production after the nanodrug treatment of hepatocellular carcinoma cells. n=3, mean ± SEM, ***P < 0.001.

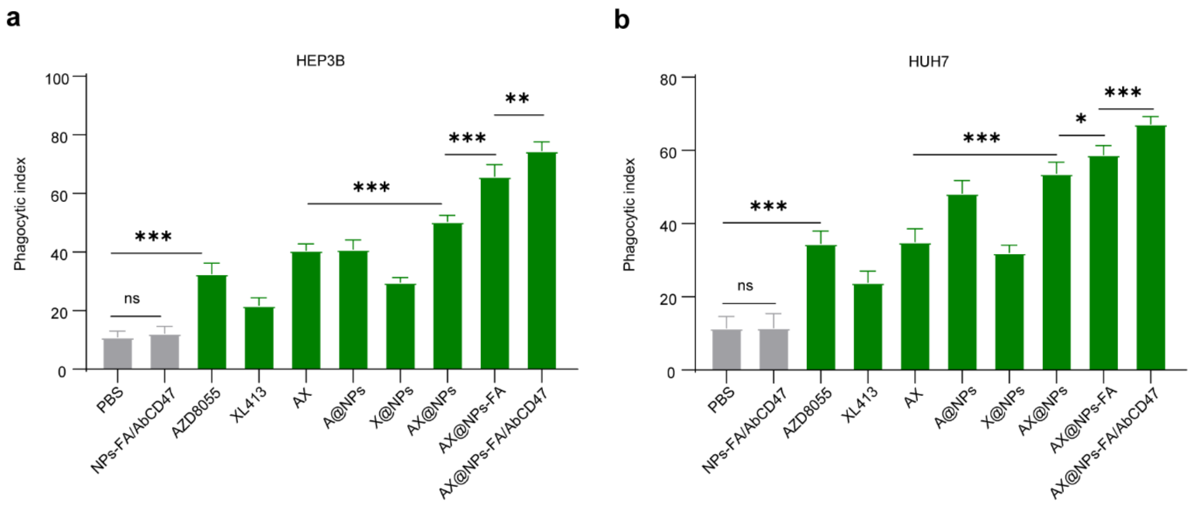

Figure S15. Statistical evaluation was performed to assess the phagocytosis of HEP3B (**a**) and HUH7 (**b**) cells by macrophages following various treatments. n=5, mean ± SEM, *p < 0.05, **p < 0.01, ***p < 0.001; p ≥ 0.05 indicates no significance (ns).

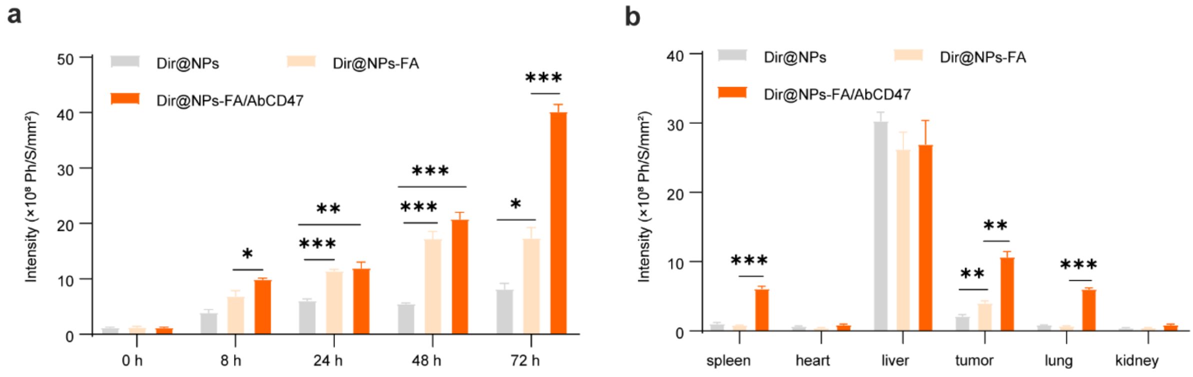

Figure S16. Quantitative analyses were performed to evaluate the fluorescence distribution of various nanomaterials (Dir@NPs, Dir@NPs-FA, Dir@NPs-FA, and Dir@NPs-FA-AbCD47) at neoplastic sites within tumour-bearing murine models at predetermined time intervals: immediately postinjection (0 h) and subsequently at 8 h, 24 h, 48 h, and 72 h (**a**). Furthermore, a comprehensive statistical assessment was conducted to investigate the spatial distribution of fluorescence within both tumour tissues and essential organs (heart, liver, spleen, lungs, and kidneys) 72 hours postinjection (**b**). n=3, mean ± SEM, *$p < 0.05$, **$p < 0.01$, ***$p < 0.001$.

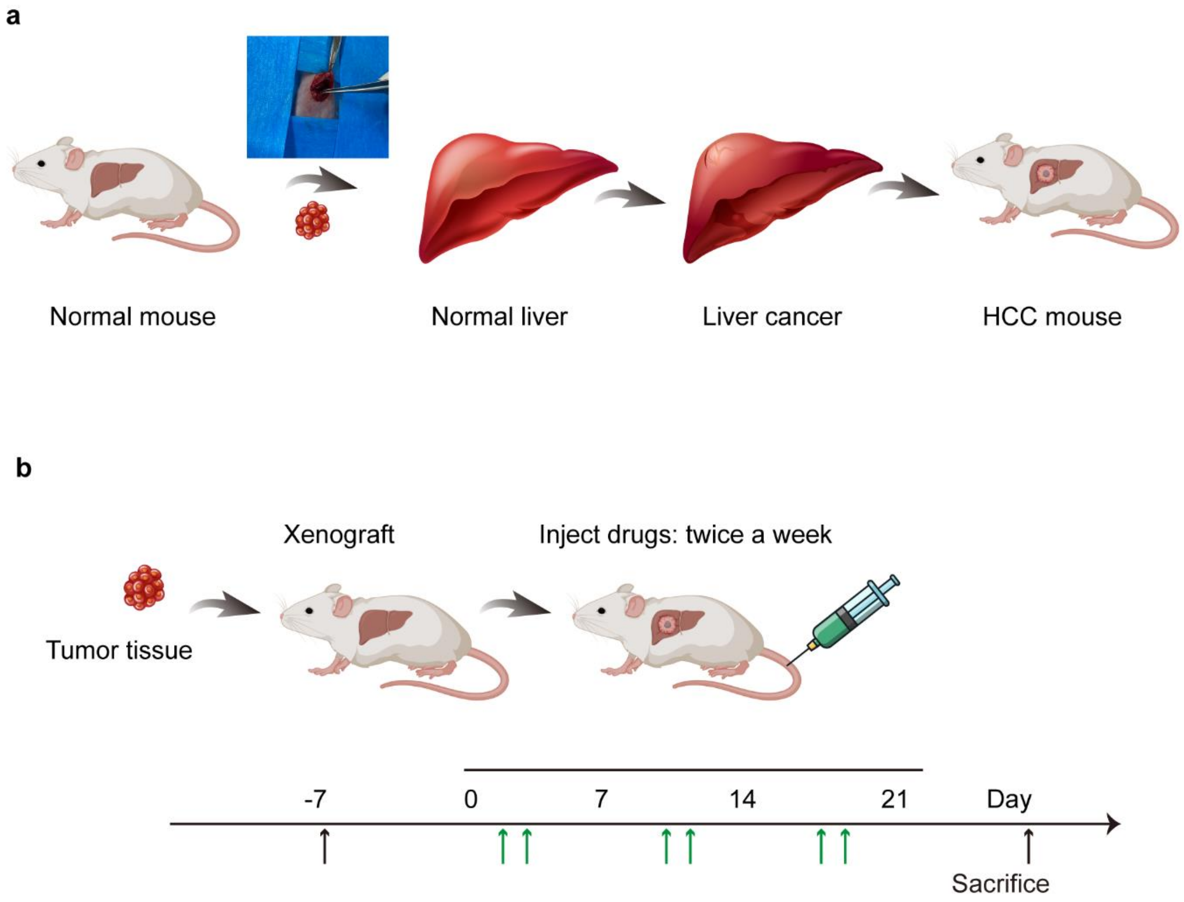

Figure S17. a. Schematic diagram of liver cancer in situ in mice (BALB/c nude: HUH7; C57BL/6: Hepa1-6). **b.** Schematic diagram of the drug administration cycle in mice (BALB/c nude: HUH7; C57BL/6: Hepa1-6).

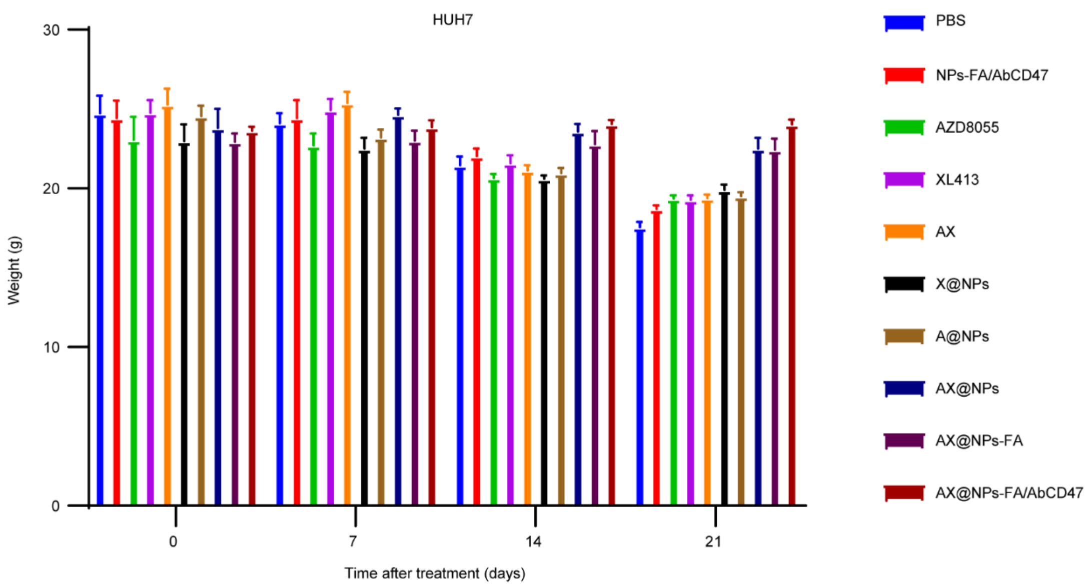

Figure S18. Changes in the body weights of HUH7 tumour-bearing nude mice after different treatments.

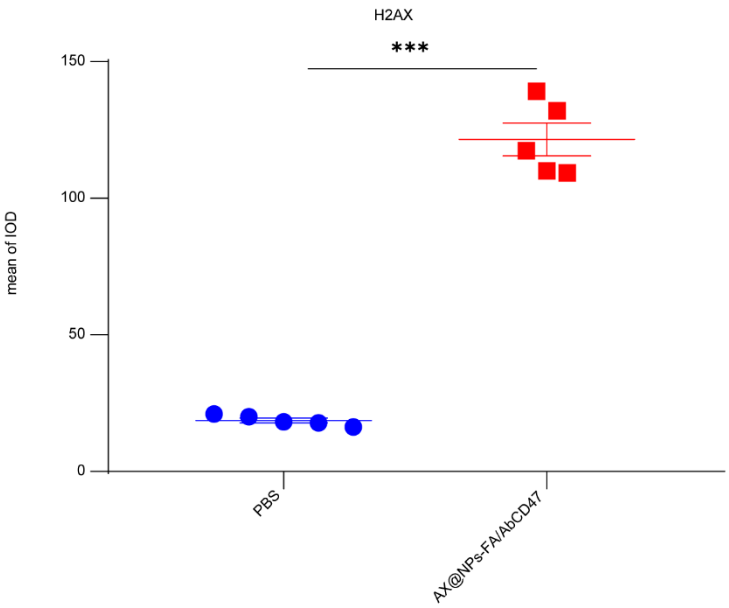

Figure S19. The statistical results of H2AX expression in various treatment groups. n=5, mean ± SEM, ***$P < 0.001$.

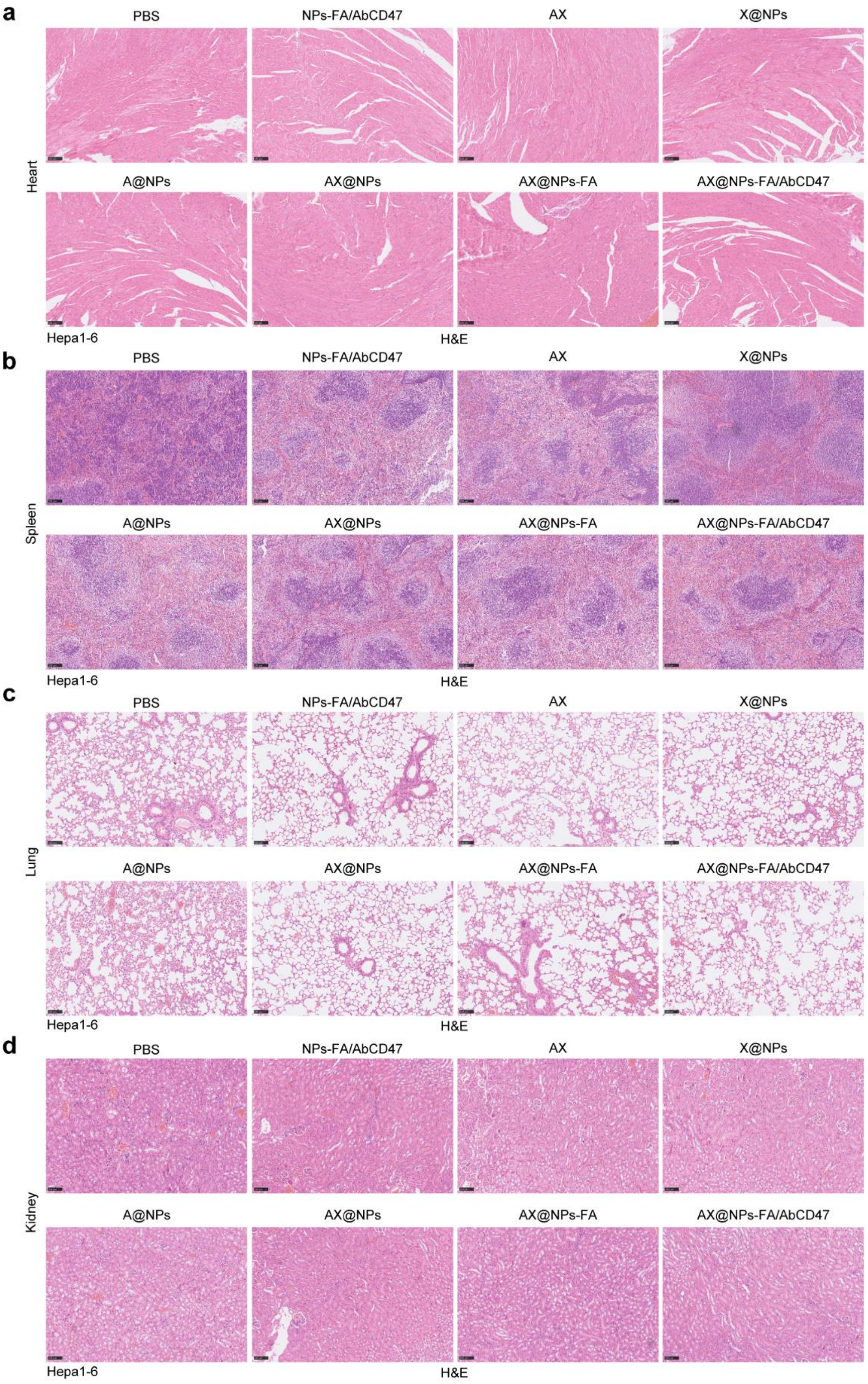

Figure S20. Treatments were essentially nontoxic to the vital organs of Hepa1-6 tumour-bearing C57BL/6 mice. **a.** H&E results for the heart; **b.** H&E results for the spleen; **c.** H&E results for the lungs; **d.** H&E results for the kidneys.
